# High-Dose Voclosporin Protects Against Acute Kidney Injury via Regnase-2-Mediated NGAL MRNA Decay

**DOI:** 10.3390/ijms27073150

**Published:** 2026-03-30

**Authors:** Kazuhiro Hasegawa, Yusuke Sakamaki, Masanori Tamaki, Sumiyo Yamaguchi, Shinji Miyakami, Chihiro Okinari, Miho Tada, Makoto Otsuka, Masanori Minato, Shu Wakino

**Affiliations:** 1 Department of Nephrology, Tokushima University Graduate School of Biomedical Sciences, 3-18-15 Kuramoto-Cho, Tokushima 770-8503, Japan; 2 Department of Nephrology and Endocrinology, National Defense Medical College, Tokorozawa 359-8513, Japan

**Keywords:** Inmt, voclosporin, Regnase-2

## Abstract

Acute kidney injury (AKI) is a major complication of lupus nephritis and kidney transplantation, inevitably causing ischemia–reperfusion (I/R) injury. We previously confirmed that high-dose voclosporin induces drug nephropathy through aberrant peroxisome accumulation. The latter induces increased renal indole-3-aceticT acid (IAA) production due to the decreased expression of the IAA-degrading enzyme indolethylamine N-methyltransferase (INMT). Conversely, *INMT* overexpression prevents this nephropathy, suggesting that high-dose voclosporin could enable a novel therapeutic approach. This prompted us to test whether *INMT* overexpression with high-dose voclosporin could avert nephrotoxicity and protect against I/R injury. *Inmt*-overexpressing mice treated with high-dose voclosporin exhibited absence of peroxisomal abnormalities and resistance to I/R injury. RNA sequencing revealed the downregulation of tubular injury markers NGAL (*Lcn2*) and KIM-1 (*Havcr1*) concurrent with significant cytokine suppression. Mechanistic analysis revealed the robust induction of Regnase-2, an mRNA decay factor, which directly targeted stem–loop structures within the 3′ untranslated region of *Lcn2* and *Havcr1*, thereby promoting their degradation in proximal tubular cells. Importantly, Regnase-2 knockdown mice showed *Lcn2* upregulation, mitochondrial dysfunction, and peroxisomal abnormalities culminating in AKI, underscoring its renal protective effects. High-dose voclosporin under *Inmt* overexpression promoted Regnase-2-mediated mRNA decay to suppress tubular injury. This protective effect extended beyond I/R to rhabdomyolysis- and lipopolysaccharide-induced AKI to prevent nephropathy. Our findings demonstrate the potential transformative therapeutic approach of administering high-dose voclosporin to promote the prophylactic effect of Regnase-2 augmentation against AKI in both native and transplanted human kidneys.

## 1. Introduction

Voclosporin (Voc), a second-generation calcineurin inhibitor (CNI), has demonstrated significant clinical efficacy and is approved for the treatment of lupus nephritis [1]. Previously, we investigated its nephrotoxic mechanism and found that high-dose Voc induces the accumulation of indole-3-acetic acid (IAA) in proximal tubules [2]. Excessive IAA deposition induces peroxisomal alterations and tubular injury, along with increased NGAL and KIM-1 levels, which function not only as biomarkers but also as pathogenic mediators.

Indolethylamine N-methyltransferase (Inmt), an enzyme that detoxifies IAA, has recently been identified as a critical nephroprotective factor. Proximal tubule-specific transgenic mice overexpressing Inmt exhibited resistance to voclosporin-induced injury by resisting peroxisomal damage and hindering NGAL and KIM-1 expression [2]. These findings led to the hypothesis that combining high-dose Voc therapy with Inmt might not only prevent nephrotoxicity but, paradoxically, enhance tubular resilience, even under ischemia–reperfusion injury (IRI). This is a core concept of the rationale for exploring “Voc pulse therapy.”

Clinically, patients with lupus nephritis receiving CNIs may experience IRI in diverse contexts, including vascular surgery, infection-associated hypercoagulability, and arterial thrombosis, due to antiphospholipid syndrome [3,4,5]. These scenarios underscore the need to expand strategies for preventing CNI-associated acute kidney injury (AKI) under ischemic stress [6,7,8,9].

Based on these insights, we aimed to: (i) define the mechanism of voclosporin-induced nephrotoxicity under IRI; (ii) confirm that concurrent high-dose Voc and Inmt administration can ameliorate AKI; and (iii) explore Voc pulse therapy as a novel treatment paradigm with potential kidney transplantation applications.

## 2. Results

**Distinctive peroxisomal signature of Voc plus IRI**

**Figure 1:**
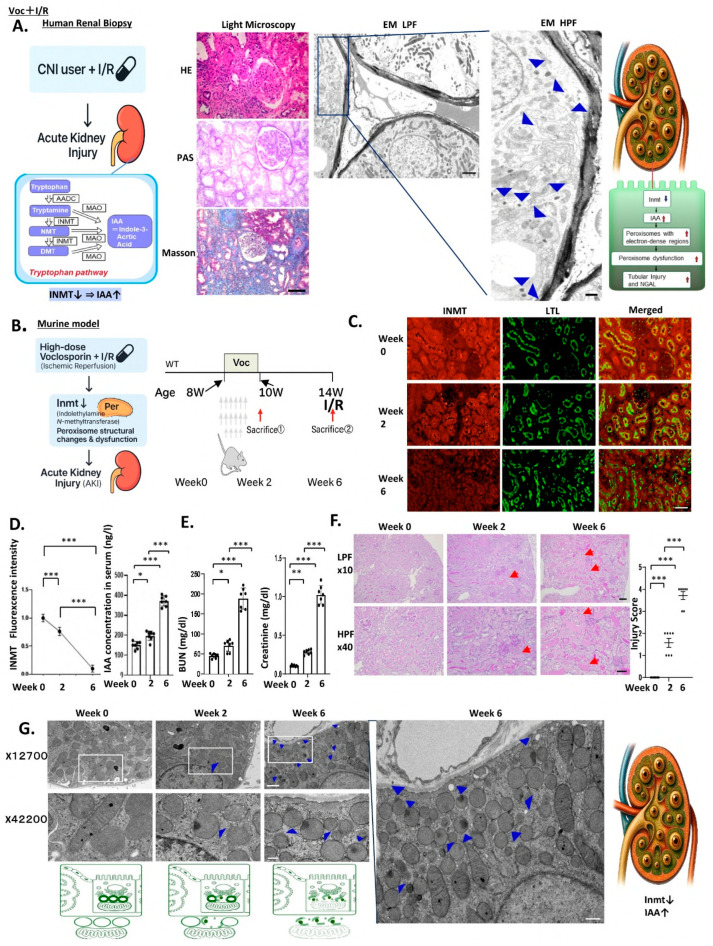
Calcineurin Inhibitors combined with ischemia/reperfusion (I/R) markedly increased the formation of “eye-like” peroxisomes associated with acute kidney injury (AKI). (**A**) Representative case of AKI in a human patient receiving CsA during renal I/R injury. Histological findings (hematoxylin–eosin, periodic acid–Schiff, and Masson trichrome staining) are confirmed by light and electron microscopy (low-power field [LPF], high-power field [HPF]), and shown by schematic illustration. It presents one of five calcineurin inhibitor-induced AKI cases summarized in Table S2, with similar ultrastructural findings observed in other patients. AADC, aromatic amino acid decarboxylase; MAO, monoamine oxidase; NMT, N-methyltryptamine; DMT, N,N-dimethyltryptamine. (**B**) Experimental protocol designed to investigate human voclosporin-associated AKI in an animal model. Because abnormal peroxisomal structures detected in (**A**) were not reproduced with standard doses of voclosporin, high-dose intraperitoneal voclosporin (30 mg/kg/day) was administered to 8-week-old mice for 2 weeks until 10 weeks of age. At 14 weeks of age, mice were subjected to I/R, and kidney tissues were analyzed 48 h later. (**C**) Fluorescent immunostaining of indolethylamine N-methyltransferase (INMT) at 8, 10, and 14 weeks (denoted as Weeks 0, 2, and 6). Double immunofluorescence with the proximal tubular marker Lotus tetragonolobus lectin. (**D**) Quantification of INMT fluorescence and measurement of plasma indole-3-acetic acid concentrations. (**E**) Serum BUN and creatinine levels progressively increased from week 0 to week 6, indicating a time-dependent decline in renal function during high-dose Voc treatment. (**F**) Histological evaluation of renal injury at Weeks 0, 2, and 6 by HE staining. Arrows indicate lysed tubules. (**G**) At Week 6, the striking accumulation of eyeball-like peroxisomes was observed on electron microscopy (12,700× and 42,200×; the latter representing magnification of the boxed region in 12,700×). Electron micrographs of 10 proximal tubules per kidney were randomly obtained for each mouse to evaluate proximal tubular morphometry. Blue arrowheads indicate renal tubular peroxisomes containing electron-dense regions. Scale bars: 100 μm (immunofluorescence), 50 μm (light microscopy), 500 nm (electron microscopy). All quantitative data in panels (**D**–**F**) were obtained from n = 7 mice per group. Statistical analyses were performed using one-way ANOVA with Bonferroni correction. Exact *p*-values are indicated in the figure. * *p* < 0.05; ** *p* < 0.01; *** *p* < 0.001.

A patient with lupus nephritis developed AKI following transient cardiac arrest complicated by I/R injury during cyclosporine A (CsA) treatment. Renal biopsy (Figure 1A) revealed clinical characteristics consistent with typical I/R-induced AKI.

However, electron microscopy uncovered an unexpected feature. Instead of the typical ultrastructural changes associated with I/R injury, we observed a profusion of “eyeball-like peroxisomes,” dense structures previously recognized as a hallmark of CNI-induced AKI [2]. In the present case, their abundance was striking: they were observed in multifocal clusters, creating an aberrant peroxisomal morphology with numerous dense-core structures. This unexpected accumulation highlights a distinctive CNI-related signature, superimposed upon an IRI background (Figure 1A). No methods for measuring Voc blood concentrations have been established in clinical practice. Unlike CsA or tacrolimus (Tac), which require therapeutic drug monitoring, Voc dosing is generally adjusted according to residual renal function parameters indicated by estimated glomerular filtration rate but without a routine evaluation of blood levels. We hypothesized that the induction of I/R may have increased Voc concentration, thereby exacerbating Voc-induced AKI, which in turn further increased the number of eyeball-like peroxisomes.

To examine whether this phenotype could be attributed to the profound reduction in INMT observed, we established a mouse model based on our previously reported Voc-induced AKI and induced I/R to analyze ultrastructural changes (Figure 1B). First, we constructed a Voc-induced AKI model by injecting Voc intraperitoneally at 30 mg/kg/day for 2 weeks in 8-week-old C57BL/6 mice. *Inmt* expression rates were assessed at 8, 10, and 14 weeks of age. To facilitate I/R-mediated AKI, bilateral renal ischemia was induced for 30 min, followed by reperfusion, in 14-week-old male C57BL/6 mice, after 6 weeks of Voc administration. Fluorescent immunostaining (Figure 1C) revealed a marked reduction in *Inmt* expression after 2 weeks of Voc treatment, with a further decline at 6 weeks, resulting in a near-complete loss (Figure 1D). Consistently, serum blood urea nitrogen (BUN) and creatinine levels increased progressively at 8, 10, and 14 weeks of age (Figure 1E). Histological evaluation with HE staining revealed proximal tubular injury, brush border loss, and cast formation at 2 weeks, which worsened at 6 weeks after Voc administration (Figure 1F). Electron microscopy revealed a striking increase in the number of eyeball-like peroxisomes after 6 weeks of Voc treatment, characterized by multiple protruding eyes. This phenotype was similar to that observed in Voc-induced AKI complicated by I/R in humans (Figure 1G). This marked rise in eye-like peroxisomes has not been previously reported and may represent a characteristic feature of I/R occurring during Voc treatment.

***Inmt* overexpression in tubule preservation under Voc and I/R stress**

**Figure 2:**
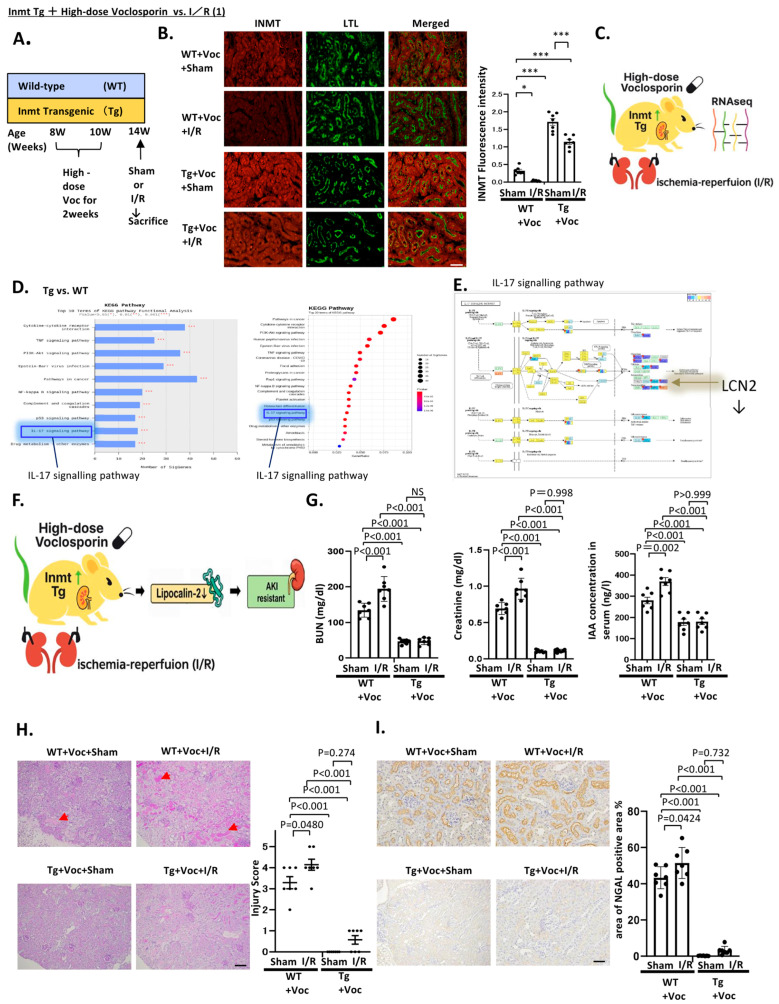
High-dose voclosporin exerted full protective capacity against superimposed ischemia/reperfusion (I/R) injury in *Inmt* transgenic mice by suppressing voclosporin nephropathy and downregulating *Lcn2*. (**A**) *Inmt* transgenic (Tg) mice with proximal tubular overexpression of *Inmt* and wild-type (WT) mice were treated with high-dose voclosporin (30 mg/kg, intraperitoneally) for 2 weeks at 8–10 weeks of age, followed by I/R at 14 weeks (sham as control). Four groups were compared: WT+Voc+Sham, WT+Voc+I/R, Tg+Voc+Sham, and Tg+Voc+I/R. Kidneys were analyzed 24 h after I/R. (**B**) Fluorescent immunostaining of INMT was performed, with double staining for the proximal tubular marker Lotus tetragonolobus lectin. INMT fluorescence intensity was quantified. (**C**) Bulk RNA sequencing was performed to compare genome-wide differential gene expression between Tg+Voc+I/R and WT+Voc+I/R kidneys. (**D**) Kyoto Encyclopedia of Genes and Genomes (KEGG) enrichment analysis of downregulated differentially expressed genes. The x-axis indicates gene ratio, and the y-axis represents KEGG terms. Circle size represents gene count, and its color represents adjusted *p*-value. (**E**) The IL-17 signaling pathway is demonstrated within the KEGG pathways identified in (**D**). (**F**) Notably, *Lcn2* expression, encoding neutrophil gelatinase-associated lipocalin (NGAL), was markedly lower in Tg+Voc+I/R, indicating that *Lcn2* downregulation mediates the protective effect against I/R-induced acute kidney injury (AKI). (**G**) Renal functional parameters. I/R+WT+Voc mice exhibited marked increases in BUN and creatinine, whereas Inmt Tg mice maintained values comparable to sham controls, demonstrating preserved renal function under Voc+I/R stress. (**H**) Histological injury scores were quantified by hematoxylin–eosin staining. Arrows indicate lysed tubules. (**I**) NGAL immunostaining was performed and quantified. Importantly, once Voc nephropathy was suppressed in *Inmt* transgenic mice, high-dose voclosporin exerted its full protective capacity, attenuating superimposed I/R-induced AKI. Scale bars: 100 μm (immunofluorescence), 50 μm (light microscopy). All quantitative data were analyzed using two-way ANOVA followed by Tukey’s post hoc test (n = 7 per group). Exact *p*-values are indicated in the figure. * *p* < 0.05; *** *p* < 0.001; NS: not significant.

**Figure 3:**
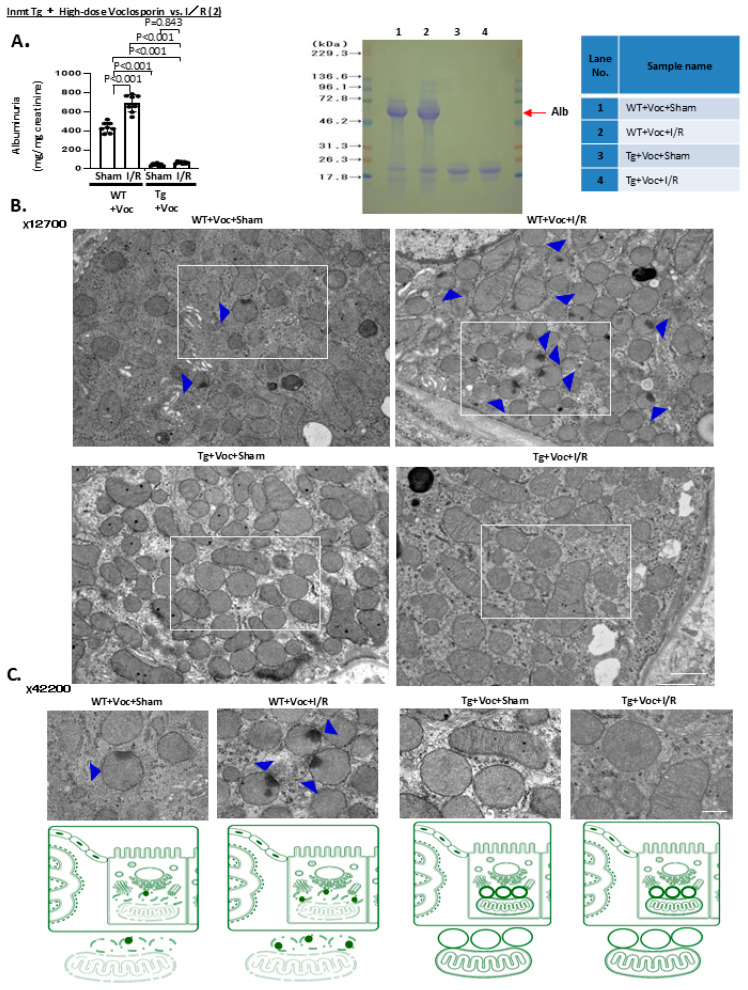
High-dose voclosporin fully suppressed ischemia/reperfusion (I/R)-induced albuminuria and eliminated “eyeball-like” peroxisome accumulation, with *Inmt* overexpression providing a permissive background. Albuminuria and abnormal peroxisomes with electron-dense regions were evident in WT+Voc+Sham mice and more pronounced in WT+Voc+I/R ones. In contrast, these changes were completely suppressed in Tg+Voc+I/R mice, in which high-dose voclosporin not only prevented I/R-induced AKI but also eliminated the emergence of eye-like peroxisomes. (**A**) Urinary albumin excretion in four groups of mice (WT+Voc+Sham, WT+Voc+I/R, Tg+Voc+Sham, and Tg+Voc+I/R). (**B**) Sodium dodecyl sulfate–polyacrylamide gel electrophoresis (SDS-PAGE) of urine samples of 14-week-old mice from each group. Samples were subjected to 15% SDS-PAGE and stained with Coomassie blue. (**C**) Representative electron micrographs from each group. White squares indicate enlarged regions; blue arrowheads represent abnormal peroxisomes with inner electron-dense structures. Illustrations below provide clarification. Kidney tissue specimens for electron microscopy were embedded in Epon epoxy resin (Hexion, Columbus, OH, USA). For morphometric evaluation, electron micrographs of 10 proximal tubules per kidney were randomly acquired from each mouse. Scale bar: 50 nm (electron microscopy). Thus, once voclosporin nephropathy was suppressed in *Inmt* transgenic mice, high-dose voclosporin therapy exerted its full protective capacity, completely preventing I/R-induced acute kidney injury and abolishing the formation of eyeball-like peroxisomes. Quantitative data in panel (**A**) were analyzed using two-way ANOVA (genotype × I/R) followed by Tukey’s post hoc test (n = 7 per group). Exact *p*-values are indicated in the figure.

We next investigated a strategy for preventing the significant decrease in *Inmt* expression in proximal tubules induced by high-dose Voc administration and I/R. Accordingly, we used transgenic (Tg) mice with proximal tubule-specific *Inmt* overexpression [2] and comparatively analyzed the changes with WT mice. Both WT and Tg mice were treated with high-dose Voc, followed by I/R or sham operation for controls. Four experimental groups were established as follows (Figure 2A):

1. Sham+WT+Voc (WT mice subjected to sham operation and treated with high-dose Voc)
2. I/R+WT+Voc (wt mice subjected to I/R and treated with high-dose Voc)
3. Sham+Tg+Voc (tg mice undergoing sham operation and treated with high-dose voc)
4. I/R+Tg+Voc (Tg mice subjected to I/R and treated with high-dose voclosporin) Immunofluorescence staining revealed that *Inmt* expression further decreased in the I/R+WT+Voc group compared with that in the Sham+WT+Voc group. In contrast, *Inmt* expression was preserved in the Sham+Tg+Voc group and, notably, in the I/R+Tg+Voc group compared with the I/R+WT+Voc group (Figure 2B). To explore genome-wide changes beyond *Inmt*, bulk RNA-seq analysis of kidneys was performed in the I/R+Tg+Voc and I/R+WT+Voc groups (Figure 2C). The expression of EGF, a growth factor involved in tubular repair, and transporters, such as Slc22a22, Slc22a28, and Slc7a13, which facilitate metabolite excretion and tubular homeostasis, were upregulated together with INMT and remained relatively stable in the I/R+Tg+Voc group. Such processes were suggestive of tubular protection (Table S3). Furthermore, the I/R+Tg+Voc group exhibited markedly lower amounts of TNF- and IFN-related inflammatory cytokines, as well as tubular injury markers NGAL (encoded by *Lcn2*) and KIM-1 (encoded by *Havcr1*) (Table S4). Gene Ontology and Kyoto Encyclopedia of Genes and Genomes (KEGG) pathway analyses further confirmed the suppression of pro-inflammatory cytokines (Figure 2D). Notably, within the KEGG pathways, multiple pro-inflammatory mediators in the IL-17 signaling cascade involving NGAL were significantly downregulated in the I/R+Tg+Voc group (Figure 2E). NGAL is a well-established marker of AKI, particularly of tubular damage. However, it also exerts direct injurious influence through mitochondrial dysfunction [10] and enhanced oxidative stress in the Fenton reaction [11,12,13]. We next evaluated the degree of AKI induced in the four groups (Figure 2F). The I/R+Tg+Voc group exhibited lower serum BUN and creatinine levels (Figure 2G), less pronounced tubular injury (Figure 2H), decreased NGAL expression (Figure 2I), and diminished urinary protein excretion (Figure 3A) compared with the I/R+WT+Voc group. The findings further align with those of RNA-seq analysis, indicating that proximal tubular overexpression of *Inmt* suppresses the I/R-provoked reduction in *Inmt* expression even under high-dose Voc administration, thereby conferring tubular protection by attenuating the expression of inflammatory cytokines and injury markers. Interestingly, the I/R+WT+Voc group exhibited a substantial increase in the number of eyeball-shaped peroxisomes on electron microscopy. This phenomenon may occur because WT mice develop AKI due to Voc nephrotoxicity, and further AKI caused by I/R induces an increase in Voc concentrations, resulting in a significant reduction in *Inmt* expression (Figure 3B). Thus, while high-dose Voc therapy can suppress I/R-induced AKI under *Inmt* overexpression, it can induce both Voc nephrotoxicity and I/R-related AKI under *Inmt* knockout.

**Reg-2 directly targeting *Lcn2* and *Havcr1* in tubular injury**

We next investigated the factor responsible for the extensive tubular cell injury observed in Tg mice. Among the candidate factors, Reg-1 is highly expressed in the kidneys, particularly in proximal tubular cells [14]. Meanwhile, other mRNA regulatory factors, such as tristetraprolin (TTP), adenine–uridine (AU)-rich element RNA-binding protein 1 (AUF1), T-cell intracellular antigen-1 (TIA-1), and up-frameshift protein 1 (UPF1), are mainly localized in immune cells, and their expression in renal proximal tubules has not been reported [15]. To verify these observations, we performed real-time polymerase chain reaction (PCR) analysis using whole kidneys harvested from 14-week-old WT mice. Consistent with previous reports, Reg-1 was easily detected, whereas other regulatory factors (TTP, AUF1, TIA-1, and UPF1) were not found expressed in kidneys (Figure S1A).

**Figure 4:**
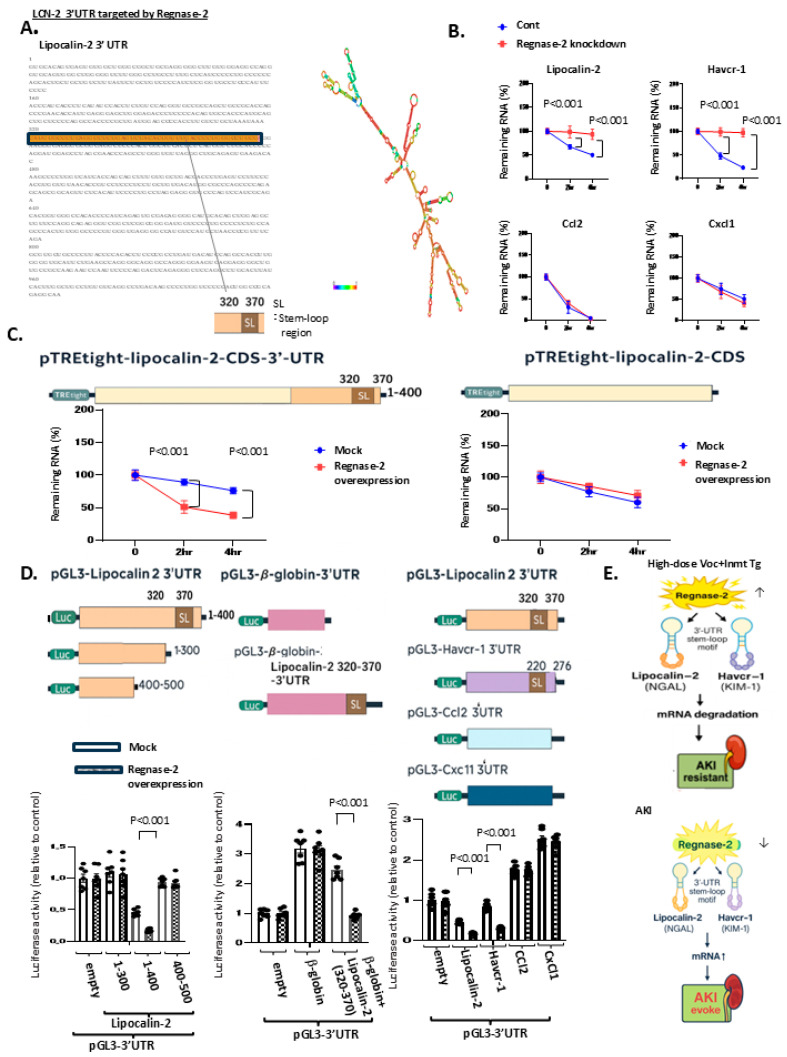
Regnase-2 mediated the protective effect of high-dose voclosporin against acute kidney injury (AKI) by destabilizing injury-related transcripts, including *Lcin2* and *Havcr1* mRNAs. (**A**) Sequence analysis of the *Lcn2* 3′ untranslated region (UTR) revealed a stem–loop structure (nucleotides 220–270, highlighted in yellow) that represents the Regnase-2 recognition motif. RNA-fold minimum free energy (MFE) prediction illustrates the secondary structure of the *Lcn2* 3′-UTR. (**B**) NGAL and KIM-1 are not only injury markers but also pathogenic mediators that facilitate proximal tubular injury, partially through mitochondrial dysfunction and subsequent peroxisomal impairment. Proximal tubular cells isolated from Regnase-2 conditional knockout (CKO) and control mice were cultured and treated with actinomycin D (2 μg/mL) to block transcription. Quantitative real-time PCR analysis revealed that *Lcn2* and *Havcr1* mRNAs were markedly stabilized in CKO cells. Quantification relative to *Gapdh* confirmed that Regnase-2 normally promotes the degradation of these transcripts. (**C**) In HEK293 Tet-off cells, co-transfection experiments demonstrated that Regnase-2 reduced *Lcn2* mRNA levels only when the 3′ UTR was present, but not when only the coding sequence (CDS) was expressed, as determined by quantitative real-time PCR. This indicates that the 3′ UTR is essential for Regnase-2-mediated destabilization. (**D**) Reporter assays using luciferase constructs with different regions of the *Lcn2* 3′-UTR identified the stem–loop motif as the Regnase-2–responsive element. Similar suppression was found when 3′ UTRs of *Havcr1*, *Ccl2*, and *Cxcl1* were tested, confirming that Regnase-2 broadly targets injury-related transcripts through the 3′ UTR. (**E**) Schematic summary: Under high-dose voclosporin combined with *Inmt* transgenic expression, Regnase-2 levels were elevated, leading to the degradation of *Lcn2* and *Havcr1* mRNAs and thereby diminishing tubular injury. In contrast, during high-dose voclosporin treatment under ischemia/reperfusion-induced AKI, Regnase-2 expression was reduced, resulting in stabilization and accumulation of *Lcn2* and *Havcr1* transcripts, which promoted proximal tubular injury. Panels (**B**,**C**) were analyzed using two-tailed unpaired Student’s *t*-tests (n = 3 independent experiments per group). Panel (**D**) was analyzed using two-tailed unpaired Student’s *t*-tests (n = 7 per construct). Exact *p*-values are indicated in the figure.

Four Regnase isoforms (Reg-1 to Reg-4) have been identified [16]. We therefore compared the expression of Reg isoforms in Tg mice (Figure S1B). Reg-1 and Reg-2 expression was detected in WT mice, with Reg-2 being markedly elevated in proximal tubule-specific *Inmt* Tg mice with high-dose Voc administration. Based on this, we hypothesized that Reg-2 mediates *Lcn2* mRNA degradation. Furthermore, less pronounced Reg-2 expression can contribute to increased *Lcn2* expression through impaired mRNA decay. Consistently, we identified a putative stem-loop motif within mouse nucleotides 320–370. RNA-fold analyses of the 3′ untranslated region (UTR) of *Lcn2* indicate the presence of this structure that may serve as a potential Reg-2 binding site (Figure 4A). Importantly, a similar stem-loop motif was also identified in the 3′-UTR of human *LCN2*, signifying that this ~20-nucleotide sequence is conserved across species and is likely critical for Reg-mediated degradation and mRNA destabilization. To prove this, we examined *Lcn2* mRNA expression under different conditions. In proximal tubular cells treated with actinomycin D, the half-life of *Lcn2* mRNA was significantly prolonged in cells derived from Reg-2 CKO mice, whereas no change was detected in *Ccl2* or *Cxcl1* mRNA, which were upregulated on RNA-seq of CKO mice (Figure 4B). These findings confirm that Reg-2 regulates the post-transcription of *Lcn2* mRNA.

To confirm whether Reg-2 directly controls *Lcn2* mRNA, we employed HEK293 Tet-off cells stably expressing a fusion protein of the tetracycline repressor and the VP-16 transcriptional activation domain. A plasmid (pTREtight-*Lcn2*-CDS-3′UTR) containing a *Lcn2* coding sequence (CDS) and 3′-UTR under the control of a tetracycline-responsive promoter (TRE) was introduced. Upon doxycycline treatment, *Lcn2* transcription was arrested, time-dependently decaying the mRNA (Figure 4C, left). *Reg-2* overexpression apparently accelerated *Lcn2* mRNA degradation. In contrast, the expression of *Lcn2* CDS lacking the 3′-UTR (pTREtight-*Lcn2*-CDS) was unaffected by *Reg-2* (Figure 4C, right).

Motif analysis revealed that the mouse *Lcn2* 3′-UTR had a stem-loop structure, spanning nucleotides 330–350, composed of an AU-rich loop (adenine–uridine-rich element, ARE) and a guanine–cytosine-rich stem, representing a canonical Regnase target site (Figure 4D). A similar sequence was identified in human *LCN2* mRNA, suggesting that a conserved ~20-nucleotide motif is critical for Reg-mediated degradation and mRNA destabilization. To confirm the functional relevance of this region, we generated luciferase reporter constructs (pGL3) including various segments of the *Lcn2* 3′-UTR. Insertion of the full-length *Lcn2* 3′-UTR (1–400) reduced luciferase activity compared with the reporter alone, and co-expression of Reg-2 further suppressed luciferase activity of pGL3-*Lcn2* 3′-UTR (1–400). Conversely, luciferase activity of pGL3-*Il6* 3′-UTR (1–300) or pGL3-*Il6* 3′-UTR (400–500) was unaffected by *Reg-2* expression.

Moreover, although luciferase activity of pGL3-*β-globin* 3′-UTR was not impacted by Reg-2, the addition of the *Lcn2* stem-loop motif (330–350) to the *β-globin* 3′-UTR conferred Reg-2 responsiveness. *Reg-2* expression also hindered the luciferase activity of a reporter containing the *Havcr1* 3′-UTR, whereas those containing the *Ccl2* or *Cxcl1* 3′-UTRs remained unchanged. These results indicate that *Lcn2* and *Havcr1* mRNAs are directly regulated by Reg-2 (Figure 4E). In contrast, *Ccl2* and *Cxcl1*, which are interferon- and TNF-responsive genes, may be secondarily upregulated in Reg-2 CKO mice as a consequence of excessive *Lcn2* and *Havcr1* production.

**Reg-2 deficiency in proximal tubules induces AKI through mitochondrial and peroxisomal dysfunction.**

To examine the physiological significance of Reg-2 in proximal tubules, we then analyzed whether proximal tubule-specific deficiency of Reg-2 (Reg-2), contributes to the development of AKI (Figure 3B). in contrast to Tg mice, we generated proximal tubule-specific Reg-2 knockout mice (Reg-2 CKO), using an AAV9 viral infection system with immunostaining-confirmed lower Reg-2 expression (Figure S1C,D). Reg-2 CKO mice exhibited AKI, accompanied by acute tubular injury (Figure S1E,F) and proteinuria (Figure S1G). Furthermore, RNA-seq analysis (Figure S1H; Tables S5 and S6) revealed the upregulation of numerous cell injury-related molecules, with a particularly highly prominent *Lcn2* expression (Table S5). The expression of NGAL, the protein encoded by *Lcn2*, was also markedly elevated in Reg-2 CKO mice (Figure S1J). Taken together, these findings demonstrate that reduced Reg-2 expression contributes to the upregulation of *Lcn2*.

We next investigated how *Lcn2* promoted by Reg-2 knockdown in proximal tubules activates tubular dysfunction and the development of AKI. RNA-seq analysis of Reg-2 CKO mice revealed decreased mRNA expression of key enzymes in mitochondrial and peroxisomal functions within the corresponding KEGG pathways. These findings were validated by immunofluorescence staining and real-time PCR.

Because *Lcn2* causes mitochondrial dysfunction (Figure S2A), we first evaluated the mitochondrial function, particularly fatty acid β-oxidation (FAO). Immunofluorescence staining of medium-chain acyl-CoA dehydrogenase, a critical enzyme for FAO, was significantly less expressed in CKO mice (Figure S2B). Furthermore, real-time PCR revealed that the expression of peroxisome proliferator-activated receptor gamma coactivator-1 alpha (PGC1α), a regulator of mitochondrial biogenesis, was also more significant in CKO mice (Figure S2C).

We next assessed the functions of peroxisomes, which play an important role in FAO, together with mitochondria. The expression of catalase (a reactive oxygen species [ROS] scavenger), acyl-CoA oxidase (a functional enzyme in peroxisomal FAO), and 70-kDa peroxisomal membrane protein (a marker of peroxisome abundance) was all significantly downregulated in CKO mice (Figure S2D,E). These mitochondrial and peroxisomal impairments led to increased ROS production, as reflected by higher 4-hydroxynonenal levels in CKO mice (Figure S2F), and enhanced apoptosis of proximal tubular cells, as revealed by TUNEL staining (Figure 2G).

Taken together, these results demonstrate the molecular mechanisms at the core of *Lcn*-mediated mitochondrial and peroxisomal dysfunction, ROS accumulation, and subsequent proximal tubular apoptosis in Reg-2 CKO mice (Figure S2H,I).

**High-dose Voc conferring broad AKI resistance via Reg-2 activation**

High-dose Voc administration in *Inmt* Tg mice, under the conditions where Voc-induced nephropathy had been prevented, led to the activation of Reg-2 and suppression of NGAL and KIM-1 expression, which collectively exerted a protective effect against I/R. These findings demonstrate that high-dose Voc, administered by pulse therapy during immunosuppression in organ transplantation, may also prevent IRI during transplantation.

**Figure 5:**
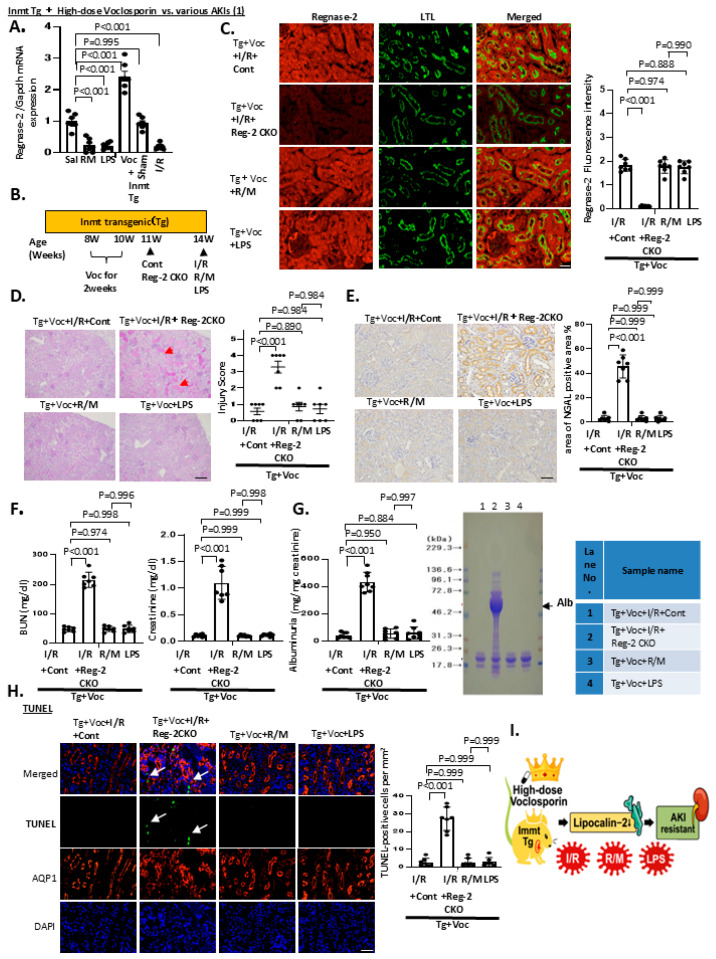
High-dose voclosporin mitigates diverse acute kindey injury (AKI) phenotypes in *Inmt* transgenic (Tg) mice through Regnase-2-dependent mechanisms. (**A**) Real-time polymerase chain reaction analysis of renal *Regnase-2* mRNA expression in 14-week-old wild-type (WT) mice subjected to rhabdomyolysis (R/M), lipopolysaccharide (LPS), or ischemia–reperfusion (I/R) injury for 24 h. High-dose voclosporin (30 mg/kg/day, i.p. for 2 weeks at 8–10 weeks of age) markedly enhanced Regnase-2 expression, whereas R/M, LPS, and I/R reduced its levels. (**B**) To determine whether voclosporin maintains Regnase-2 expression under AKI stress, 14-week-old *Inmt* transgenic mice treated with high-dose voclosporin were subjected to I/R, R/M, or LPS, and renal phenotypes were evaluated after 48 h. In Tg+Voc+I/R mice, Regnase-2 knockdown was induced by tail-vein injection of AAV9-shRNA at 10 weeks of age (control shRNA as mock) to establish Regnase-2 conditional knockout (Reg-2 CKO) mice. Regnase-2 knockdown eliminated the protective effect of voclosporin, confirming the drug interaction. (**C**) Four groups were comparatively analyzed: Tg+Voc+I/R+control shRNA, Tg+Voc+I/R+Reg-2 CKO, Tg+Voc+R/M, and Tg+Voc+LPS. For consistency, control shRNA AAV9 was also administered at 10 weeks in the latter two groups. Representative immunofluorescence images of kidney cryosections (Regnase-2 [green] and Lotus tetragonolobus lectin [red]) are shown for each group at 14 weeks. (**D**) Representative hematoxylin–eosin-stained kidney sections from WT and Tg mice in voclosporin-induced AKI (arrows indicate lysed tubules). Right panel: quantitative tubular damage scores. Scale bar: 100 μm. (**E**) NGAL immunostaining. (**F**) Serum BUN and creatinine levels. Reg-2 CKO mice showed significantly elevated BUN and creatinine compared with control shRNA mice, indicating loss of renal protection and development of AKI. (**G**) Urinary albumin excretion in the four groups at 14 weeks. Sodium dodecyl sulfate–polyacrylamide gel electrophoresis (15%) of urine specimens stained with Coomassie blue. (**H**) Apoptotic tubular cells detected by terminal deoxynucleotidyl transferase dUTP nick end labeling (arrows). (**I**) Schematic illustration: High-dose voclosporin suppresses AKI phenotypes (I/R, R/M, LPS) by promoting Regnase-2 and reducing *Lcn2*, demonstrating that its protective effect is strictly Regnase-2 dependent. Data were analyzed by one-way analysis of variance with Bonferroni correction. Scale bars: 100 μm (immunofluorescence), 50 μm (light microscopy), 500 nm (electron microscopy). All quantitative data in panels (**A**,**C**–**H**) were analyzed using one-way ANOVA followed by Bonferroni correction (n = 7 per group). Exact *p*-values are indicated in the figure.

We next investigated whether high-dose Voc combined with *Inmt* Tg confers resistance not only against I/R but also against other forms of AKI commonly observed during organ transplantation, particularly rhabdomyolysis (R/M) and infection/sepsis-associated AKI. In both the R/M (glycerol injection) and sepsis (LPS injection) models, Reg-2 expression was markedly decreased to levels comparable to those observed in I/R, indicating that AKI models induced by oxidative stress and ischemia are linked to Reg-2 downregulation (Figure 5A).

To determine whether the observed AKI resistance was Reg-2 dependent, we established Reg-2 CKO mice and subjected them to I/R (Figure 5B). Reg-2 expression was effectively knocked down in Reg-2 CKO mice, and the protective effect against AKI was eliminated (Figure 5C–H), confirming the positive association between AKI resistance and Reg-2 dependence.

Subsequently, we examined whether high-dose Voc therapy combined with *Inmt* Tg could prevent AKI in the R/M and LPS models, both of which exhibited pronounced Reg-2 downregulation. The treatment preserved Reg-2 levels (Figure 5C) while inhibiting AKI severity (Figure 5D), NGAL elevation (Figure 5E), increased BUN and creatinine levels (Figure 5F), proteinuria (Figure 5G), and accumulation of TUNEL-positive cells (Figure 5H). Taken together, these findings demonstrate that a high-dose Voc regimen, characterized by Voc-induced nephrotoxicity inhibited by *Inmt* Tg, exerted protective effects against multiple AKI models via Reg-2-mediated mechanisms (Figure 5I).

Notably, when AKI models, such as I/R, were established using *Inmt* Tg mice without high-dose Voc administration, no protective effects were observed (Figure S3), proving that AKI resistance was attributable to Voc itself rather than to *Inmt* expression. However, the Reg-2 activation mechanism by high-dose Voc remains unclear and warrants further studies.

**Figure 6:**
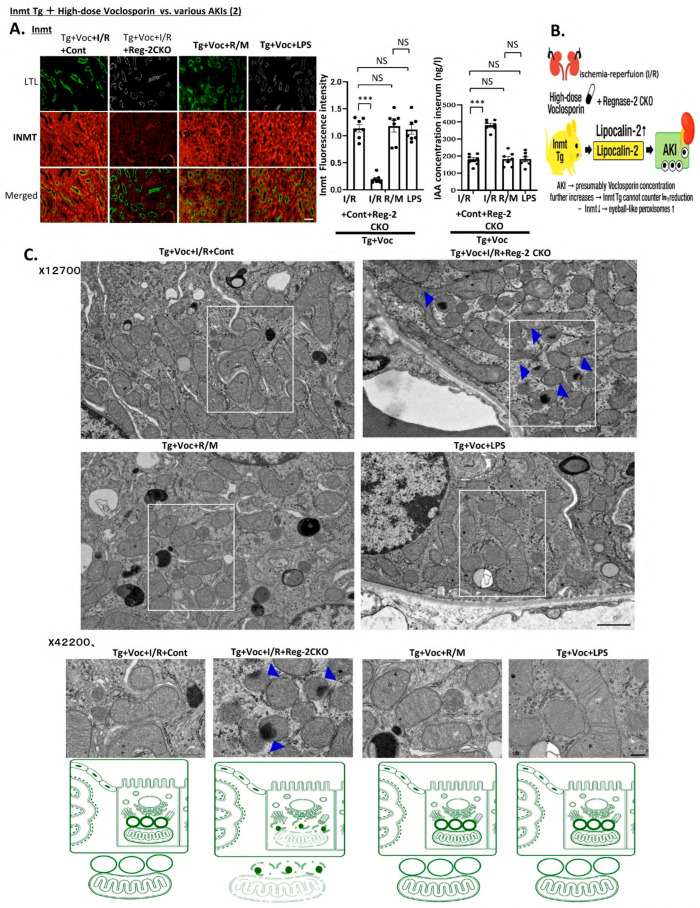
High-dose voclosporin mitigated diverse acute kidney injury (AKI) phenotypes in *Inmt* transgenic mice, but Regnase-2 knockout negated the protective effect and induced peroxisomal abnormalities. (**A**) Representative immunofluorescence images of kidney cryosections from each experimental group corresponding to Figure 5 (four groups of mice) at 14 weeks of age, while showing indolethylamine N-methyltransferase (INMT; green) and Lotus tetragonolobus lectin (LTL; red). Mean serum indole-3-acetic acid (IAA) concentrations in each group are also shown. (**B**) Schematic illustration: High-dose voclosporin mitigated diverse AKI phenotypes in *Inmt* transgenic mice by promoting Regnase-2, whereas Regnase-2 knockout eliminated this protective effect, reduced *Inmt* expression, increased IAA levels, and triggered the striking emergence and expansion of abnormal “eyeball-like” peroxisomes. (**C**) Representative electron micrographs for each mouse group, with corresponding schematic illustrations below (white squares indicate enlarged regions). Enlarged electron micrographs highlighting abnormal peroxisomes. Blue arrowheads denote inner electron-dense regions. Kidney tissue specimens were embedded in Epon epoxy resin (Hexion, Columbus, OH, USA) for electron microscopy. Ten proximal tubules per kidney from each mouse were randomly imaged for morphometric evaluation. All groups consisted of seven mice. Data were analyzed by one-way analysis of variance with Bonferroni correction. Scale bars: 100 μm (immunofluorescence), Scale bars: 500 nm (electron microscopy). Thus, the protective effect of high-dose voclosporin against AKI is strictly Regnase-2 dependent, and its loss demonstrates a novel peroxisomal pathology. All groups consisted of seven mice. Quantitative data in panel (**A**) were analyzed using one-way ANOVA followed by Bonferroni correction (n = 7 per group). Exact *p*-values are indicated in the figure. *** *p* < 0.001; NS: not significant.

Interestingly, in the I/R group of Reg-2 CKO mice cross-bred with high-dose Voc-treated *Inmt* Tg mice, not only was AKI not suppressed, but *Inmt* expression was markedly lower, whereas IAA levels increased (Figure 6A), potentially due to higher systemic or renal Voc concentrations secondary to AKI. Electron microscopy revealed a notable increase in eyeball-like peroxisomes, a morphological hallmark previously associated with Voc-induced nephropathy (Figure 6B,C).

**Figure 7:**
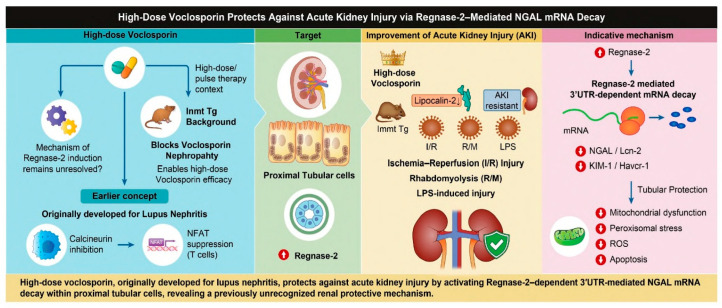
High-dose Voclosporin Protects Against Acute Kidney Injury via Regnase-2-Mediated NGAL mRNA Decay. Schematic representation of the renal protective mechanism of high-dose voclosporin in acute kidney injury (AKI). High-dose voclosporin, originally developed for lupus nephritis, is administered under an Inmt transgenic (Tg) background that prevents voclosporin-induced nephropathy and enables therapeutic efficacy. In proximal tubular cells, voclosporin upregulates Regnase-2, a ribonuclease that promotes 3′UTR-dependent mRNA decay of NGAL (Lcn-2) and KIM-1 (Havcr-1), leading to reduced expression of these injury markers. This mechanism confers resistance to multiple AKI models, including ischemia–reperfusion (I/R), rhabdomyolysis (R/M), and LPS-induced injury, and is associated with decreased mitochondrial dysfunction, peroxisomal stress, reactive oxygen species (ROS), and apoptosis. The induction mechanism of Regnase-2 remains unresolved.

Collectively, these findings establish Reg-2 as a novel and essential regulator of proximal tubular injury, directly suppressing pathogenic factors, such as *Lcn2* and *Havcr1*, while secondary cytokine changes (*Ccl2*, *Cxcl1*) indicated downstream consequences. Importantly, we demonstrate for the first time that high-dose Voc therapy, when Voc-induced nephrotoxicity is neutralized by *Inmt* expression, robustly activates Reg-2 and thereby confers protection across diverse AKI models, including I/R, R/M, and sepsis-associated AKI. This discovery highlights both the therapeutic potential of Voc pulse therapy in transplantation and the previously unrecognized function of Reg-2 as a gatekeeper of tubular injury (Figure 7).

**Renal functional parameters**

Across all experimental models, serum BUN and creatinine consistently tracked the severity of renal injury and closely aligned with the molecular and ultrastructural phenotypes observed in this study.

In the time-course model of high-dose Voc (Figure 1), both parameters increased progressively from week 0 to week 6, indicating a gradual decline in renal function that paralleled reduced Inmt expression, elevated IAA, worsening tubular injury, and accumulation of eyeball-like peroxisomes.

In the Voc+I/R four-group comparison (Figure 2), I/R+WT+Voc mice showed marked renal dysfunction, whereas Inmt Tg mice maintained BUN and creatinine levels comparable to sham controls, consistent with reduced tubular injury, decreased NGAL expression, and absence of peroxisomal abnormalities.

In Reg-2 conditional knockout mice (Figure 5), the protective effect of high-dose Voc was lost, and BUN and creatinine increased significantly, demonstrating that Reg-2 is essential for maintaining renal function under Voc+I/R stress.

Finally, in R/M and LPS models (Figure 5), high-dose Voc+Inmt Tg preserved Reg-2 expression and prevented elevations in BUN and creatinine, indicating that Reg-2-dependent renal protection extends across multiple forms of AKI.

Collectively, these findings demonstrate that BUN and creatinine provide robust and quantitative functional readouts that reinforce the molecular and ultrastructural signatures described throughout the study.

## 3. Discussion

Pulse therapy with CNIs has long been avoided because of the prohibitive risk of nephrotoxicity posed by rapid drug elevation [17]. Our study overturns this dogma by demonstrating that high-dose Voc pulse therapy becomes feasible when administered under *Inmt* overexpression to completely prevent Voc nephropathy in mice [2]. This strategy not only neutralizes Voc toxicity but also converts Voc into a protective agent, thereby suppressing multiple forms of AKI, including I/R, R/M, and LPS-induced sepsis, by activating Reg-2 and suppressing NGAL and KIM-1. Because these AKI types frequently complicate organ transplantation under CNI therapy, our findings expand the potential applicability of Voc in transplantation medicine.

Clinically, we observed a patient with lupus nephritis receiving Voc, who experienced cardiac arrest and subsequent renal I/R, resulting in severe AKI. Electron microscopy revealed massive accumulation of “eyeball-like peroxisomes,” a distinctive hallmark of Voc nephropathy [2,18], which was accurately reproduced in our mouse model (Figure 1). This concordance underscores the clinical relevance of our findings.

In *Inmt* Tg mice, Voc+I/R nephropathy was completely prevented, suppressing *Lcn2* elevation (Figure 2 and Figure 3). Because LCN2 acts not only as a marker but also as a mediator of mitochondrial dysfunction [10,19], its suppression likely indicates genuine attenuation of IRI. Mechanistic analysis revealed the robust induction of Reg-2, which has not been previously described in renal biology. The loss of Reg-2 provoked AKI with increased LCN2 production, mitochondrial dysfunction, and peroxisomal abnormalities (Figures S1 and S2).

Previous studies have implicated Reg-2 in diverse extrarenal contexts, such as in the suppression of neuroinflammation and regulation of immune homeostasis [20,21]. Studies involving liver disease models have shown that Reg-2 deficiency promotes bile duct proliferation, steatosis, fibrosis, and tumorigenesis [22]. In contrast, renal functions of Reg-1 have been partially elucidated in proximal tubular inflammation [23] and in clear cell renal cell carcinoma development [24]. However, the role of Reg-2 in renal function remains unexplored. The current study establishes Reg-2 as a determinant of tubular injury that directly degrades *Lcn2* and *Havcr1* mRNAs, thereby inhibiting mitochondrial and peroxisomal damage.

Although NGAL and KIM-1 are regarded as sensitive biomarkers of tubular injury [25,26], accumulating evidence indicates their role as pathogenic mediators. NGAL induces mitochondrial dysfunction via mTOR-dependent DRP1 activation and amplifies oxidative stress through iron-dependent pathways to promote apoptosis [10]. KIM-1 transiently facilitates the clearance of apoptotic cells in the acute phase [27] and exhibits sustained expression, which contributes to tubular injury and fibrosis [28,29,30,31]. KIM-1 activates fatty acid uptake via proximal tubular cells, thereby driving progressive diabetic kidney disease [32]. These findings support the concept that NGAL and KIM-1 are not merely passive biomarkers but are also active drivers of injury, underscoring the significance of our finding that Reg-2 directly degrades their mRNAs to preserve mitochondrial and peroxisomal integrity.

Bioinformatic and reporter assays have confirmed that Reg-2 directly targets stem-loop motifs within the 3′ UTRs of *Lcn2* and *Havcr1* (Figure 4). Conversely, Reg-2 downregulation was consistently detected across ischemia-driven AKI models (Figure 5 and Figure 6). Thus, Reg-2 emerges as a master regulator of tubular injury that directly mediates pathogenic mechanisms while orchestrating mitochondrial and peroxisomal protection.

This study has several limitations. The therapeutic efficacy of Voc in lupus nephritis remains untested. Furthermore, the mechanism of Reg-2 induction by Voc is unknown, and Voc concentrations were not measured. Another limitation is that Reg-2 CKO was performed only in the Voc+*Inmt* Tg+I/R model, but not in the R/M or LPS ones. Although these latter models lacked direct genetic validation, the preservation of Reg-2 expression in Voc+*Inmt* Tg mice subjected to I/R, R/M, and LPS indirectly supports the conclusion that the protective effects of Voc are Reg-2 dependent across diverse AKI types. Although the protective effects of high-dose Voc against AKI may involve NFAT-independent targets [33], the specific Reg-2 upregulation mechanism of Voc specifically remains unclear.

Future research directions include investigations into the protective effects of Voc+*Inmt* Tg against CsA/tacrolimus nephrotoxicity, the activation of IAA of peroxisomal abnormalities, and the therapeutic harnessing of Reg-2 overexpression. Furthermore, establishing Reg-2 CKO mice using Flox/Cre or tamoxifen-inducible systems may be informative, as constitutive deletion can risk embryonic lethality, whereas inducible models can clarify its role in adult kidneys under stress. Likewise, pharmacologic INMT activators would facilitate clinical application of high-dose Voc with INMT augmentation. Translationally, evaluation of INMT expression or IAA levels can serve as biomarkers for predicting Voc nephropathy risks; therefore, pharmacologic activation of Reg-2 represents a broad therapeutic avenue.

The present study emphasizes the translational potential of Voc pulse therapy in clinically relevant AKI models. This approach is potentially valuable in other nephrotoxic settings beyond cytotoxic models involving cisplatin or HgCl_2_ therapies. The latter further serves as an exciting avenue for future investigations that would potentially reveal distinct mechanisms of tubular injury. Our evaluation of I/R, LPS, and R/M models is primarily centered around ischemic tubular injury, which is frequently encountered under CNI therapy. CNI nephrotoxicity is often encountered during transplantation accompanied by IRI [34,35,36,37]. Sepsis-associated AKI [38,39,40,41] is a major cause of morbidity in immunosuppressed patients, while rhabdomyolysis-induced AKI [42,43,44,45] occurs in transplant recipients and critically ill patients, which is sometimes exacerbated by drug interactions, such as CNI–statin co-therapy. By focusing on these ischemia-dominant AKI types, we underscore the translational importance of Voc pulse therapy.

Beyond the acute setting, further studies should clarify whether Voc pulse therapy effectively mitigates chronic injury and fibrosis and whether long-term immunosuppression remains safe. Comparative analyses involving CsA and tacrolimus, where pulse therapy has been historically avoided, will further highlight the novelty of our approach.

Finally, although histological assessment of cytokines can provide additional information, the central focus of our study is the identification of Reg-2 as a direct post-transcriptional regulator of NGAL and KIM-1. These molecules are not merely biomarkers but also pathogenic mediators of mitochondrial and peroxisomal injuries. By demonstrating the direct degradation by Reg-2 of their mRNAs, we have established a mechanistic link between RNA decay and organelle protection. Thus, our findings highlight a novel molecular axis of tubular injury control, distinct from known general cytokine modulation mechanisms. Pathway analyses in this study were performed using the KEGG database, a widely used reference resource for gene and protein annotation [46,47].

Despite these limitations, our findings provide important mechanistic insight into how voclosporin and INMT-dependent pathways modulate tubular stress responses and influence susceptibility to acute kidney injury. These insights establish a conceptual basis for future therapeutic strategies that harness this protective axis.

## 4. Materials and Methods

More details on the methodology are provided in the Supplemental Material section.

Transgenic and conditional knockout mice

C57BL/6J mice were purchased from CLEA Japan, Tokyo, Japan. The breeding details for all mice used in the study are outlined in the Supplemental Material. The mice used included the following: proximal tubule-specific Regnase-2 (Reg-2) conditional knockout (CKO) mice, Inmt transgenic mice (Inmt Tg) overexpressing Inmt in proximal tubules and described previously [2], and control mice.

Voc administration

Mice were acclimatized through the 3-day administration of 3% ethanol in sunflower oil solvent. Subsequently, Voc (30 mg/kg) was administered intraperitoneally daily for 2 weeks (n = 8 per group) from 8–10 weeks of age. Controls received only the solvent. To minimize cage effects, treatments were given in mixed cages. The dosage used was as described previously [2].

Mouse models of AKI

Fourteen-week-old male C57BL/6 mice underwent bilateral renal I/R for 30 min to induce AKI [6]. Rhabdomyolysis was induced by intramuscular injection of 50% glycerol (7.5 mL/kg) or saline [7]. Septic AKI was induced by intraperitoneal injection of lipopolysaccharide (LPS; Escherichia coli O111:B4; Sigma-Aldrich, St. Louis, MO, USA, 10 mg/kg) [8]. Mice were sacrificed 48 h after each induction to evaluate the changes.

RNA sequencing

RNA sequencing (RNA-seq) was performed on kidney tissues collected from:

1. Voc-treated Inmt Tg mice with I/R versus Voc-treated wild-type (WT) mice with I/R.
2. reg-2 cko mice (AAV9 shRNA) versus control shrna mice.

Raw and processed RNA-seq data have been deposited in the Gene Expression Omnibus (GEO) repository under accession numbers GSE316216 and GSE316214.

Details of anesthesia and euthanasia

Ten-week-old C57BL/6J mice (CLEA Japan), weighing approximately 25–27 g, were anesthetized with 1.5–2.0% isoflurane delivered via inhalation. At the end of the experiment, mice were euthanized under deep isoflurane anesthesia by cervical dislocation.

Human-derived specimens

Clinical data (Table S2) and peripheral blood samples were obtained from patients with AKI diagnosed per Kidney Disease Improving Global Outcomes guidelines.

Statistical analysis

Statistical analyses were conducted using Prism 8 (GraphPad, San Diego, CA, USA). Sample size was determined by power calculations in line with the 3R principle. Descriptive data are expressed as mean ± standard error of the mean, and group comparisons were performed.

## 5. Conclusions

Our study provides three major advances in understanding and therapeutically leveraging voclosporin (Voc) in kidney injury.

1. We established a feasible Voc pulse therapy strategy.
2. We demonstrated that high-dose Voc combined with Inmt overexpression suppresses diverse forms of acute kidney injury (AKI).
3. We identified regnase-2 (reg-2) as a novel regulator of tubular injury marker expression.

Together, these findings transform Voc from a drug limited by nephrotoxicity into a therapeutic agent that, when paired with INMT, activates Reg-2 to confer broad resistance to AKI. This Reg-2-dependent mechanism redefines Voc not only as a key component of lupus nephritis treatment but also as a preventive strategy for CNI-associated AKI and a potential therapeutic approach for other renal diseases, opening new possibilities in transplant and renal medicine.

## Data Availability

The datasets generated and/or analyzed during the current study are available in the Gene Expression Omnibus (GEO) under accession numbers GSE316216 and GSE316214. Both datasets were made publicly accessible on 28 March 2026. URLs:GSE316216: https://www.ncbi.nlm.nih.gov/geo/query/acc.cgi?acc=GSE316216 (accessed on 1 March 2026); GSE316214: https://www.ncbi.nlm.nih.gov/geo/query/acc.cgi?acc=GSE316214 (accessed on 1 March 2026). Further information and requests for resources and reagents are available from the corresponding author.

## Supplementary Materials

The following supporting information can be downloaded at: https://www.mdpi.com/article/10.3390/ijms27073150/s1. References [48,49,50,51,52,53,54,55,56,57] are cited in the supplementary materials.
