# Supplementary material for: High-Dose Voclosporin Protects Against Acute Kidney Injury via Regnase-2-Mediated NGAL MRNA Decay
Source: Int J Mol Sci. 2026 Mar 30;27(7):3150. doi: 10.3390/ijms27073150 (PMC13072766; doi:10.3390/ijms27073150)
Supplement: Supplementary file 1 [file ijms-27-03150-s001.zip › ijms-4210259-supplementary.pdf]

Regnase-2 CKO (1)

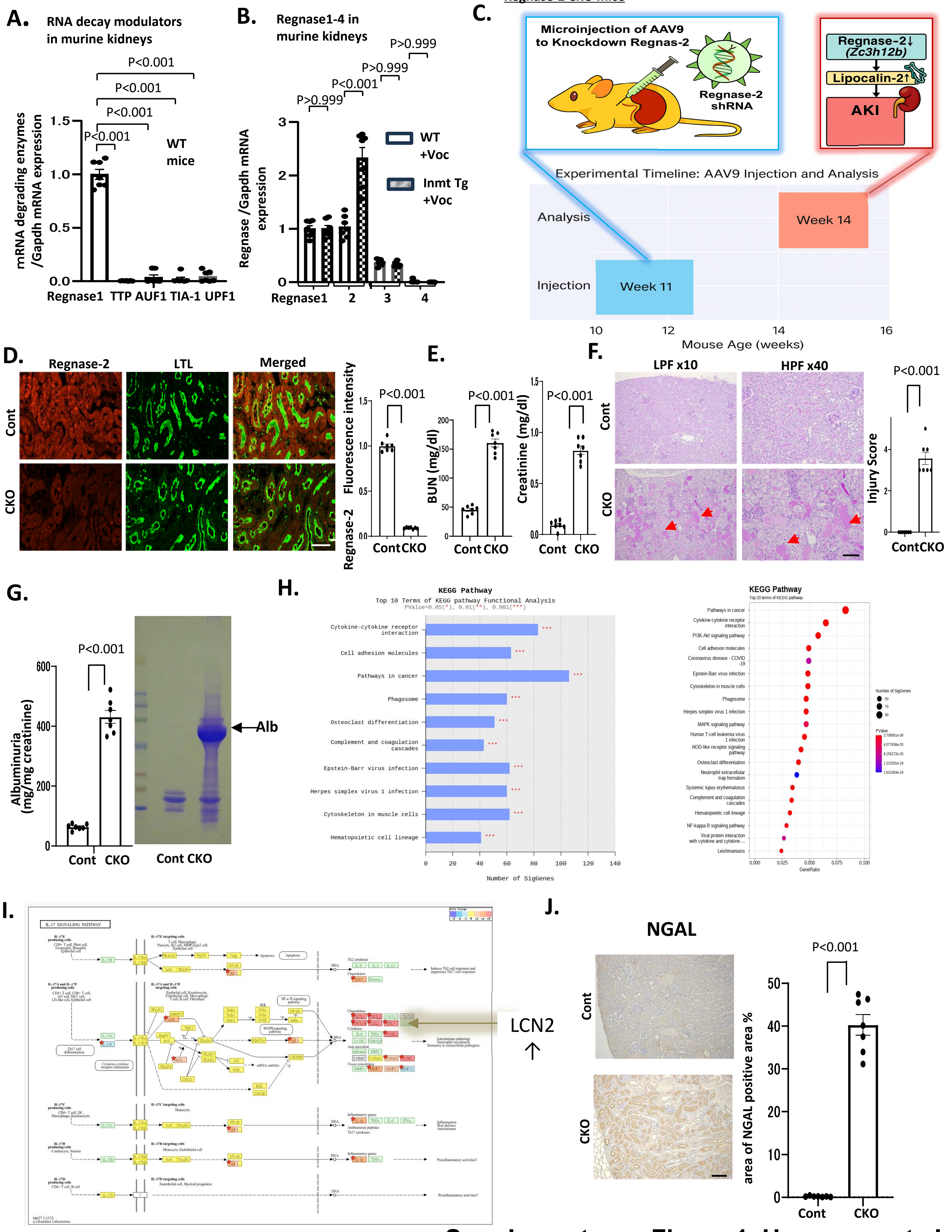

Supplementary Figure 1, Hasegawa et al.

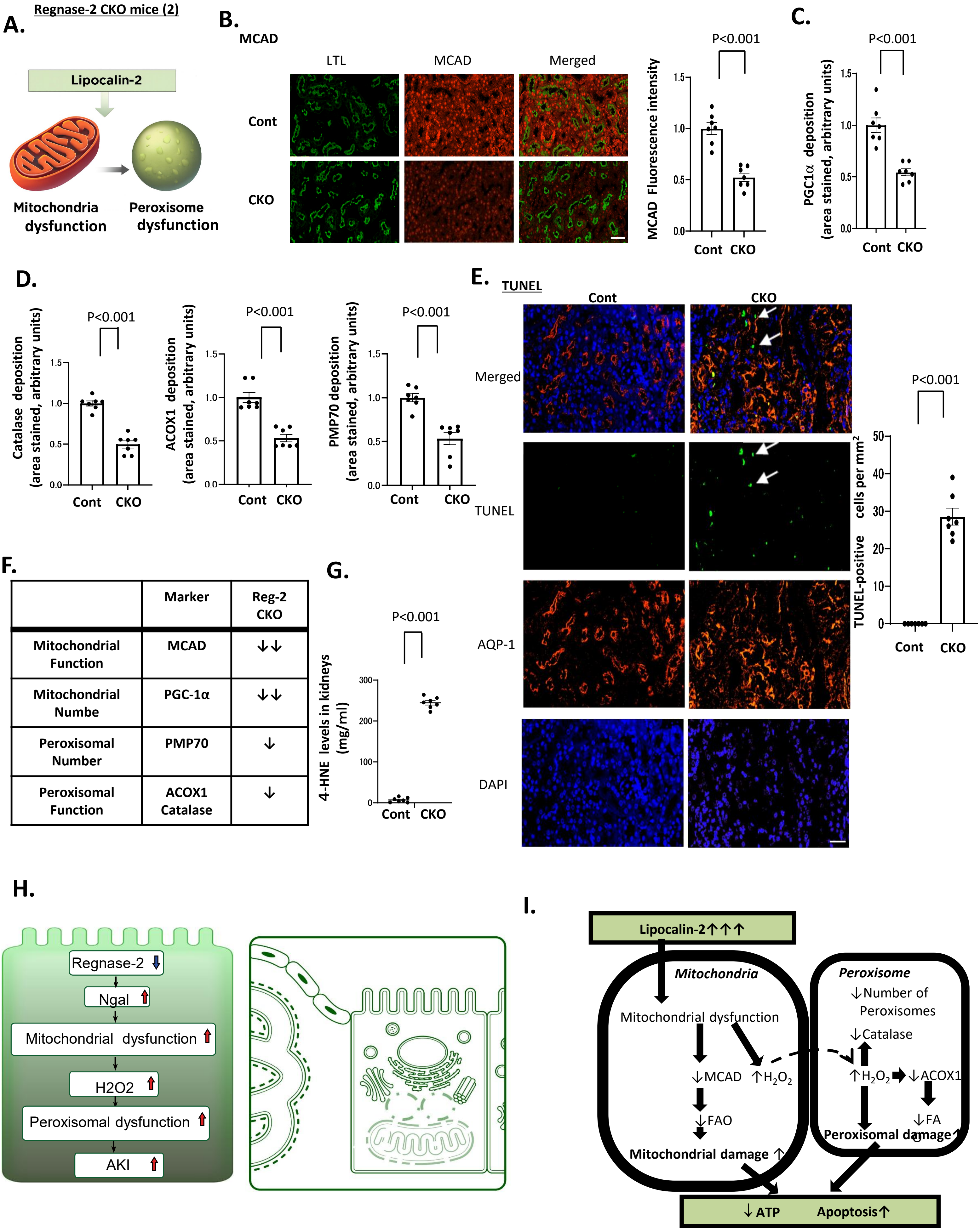

	Marker	Reg-2 CKO
Mitochondrial Function	MCAD	↓↓
Mitochondrial Numbe	PGC-1α	↓↓
Peroxisomal Number	PMP70	↓
Peroxisomal Function	ACOX1 Catalase	↓

Supplementary Figure 2, Hasegawa et al.

Inmt Tg mice without Voc administration vs. I/R

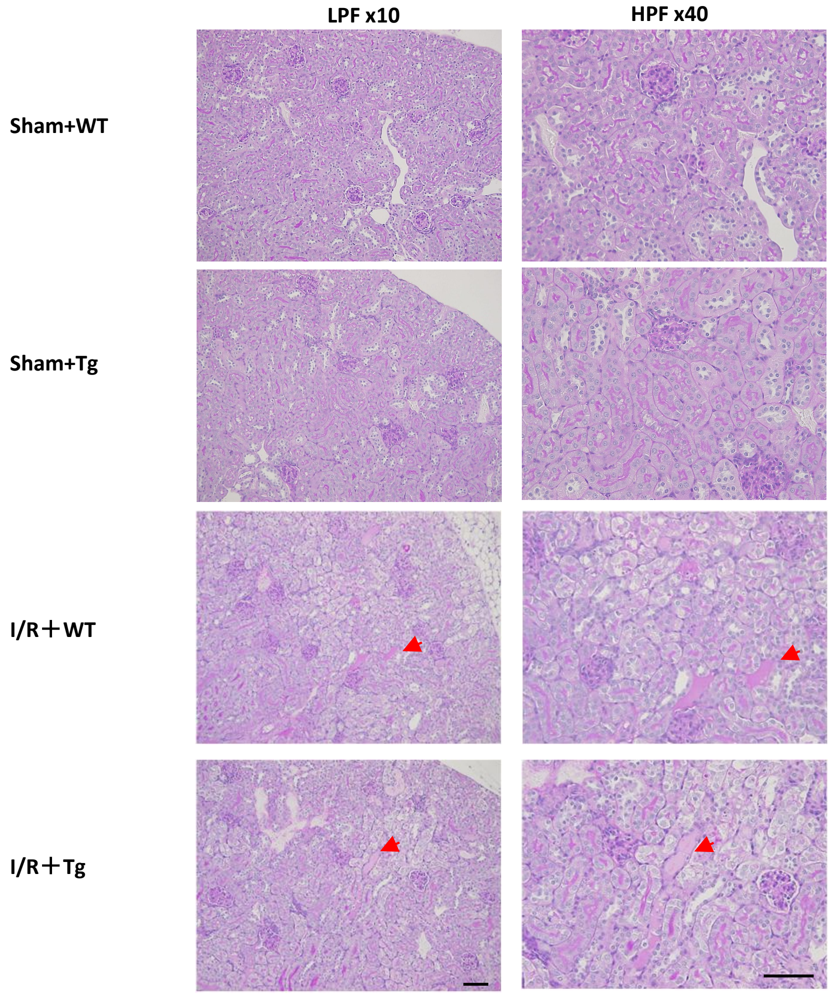

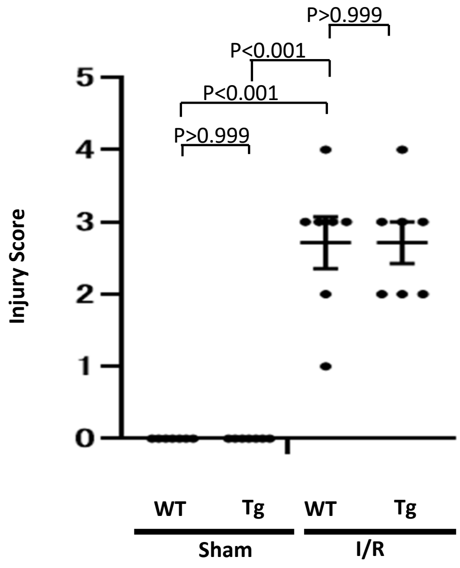

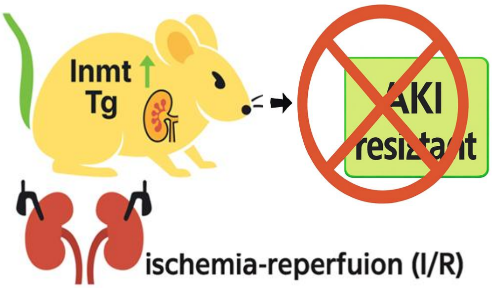

Supplementary Figure 3, Hasegawa et al.

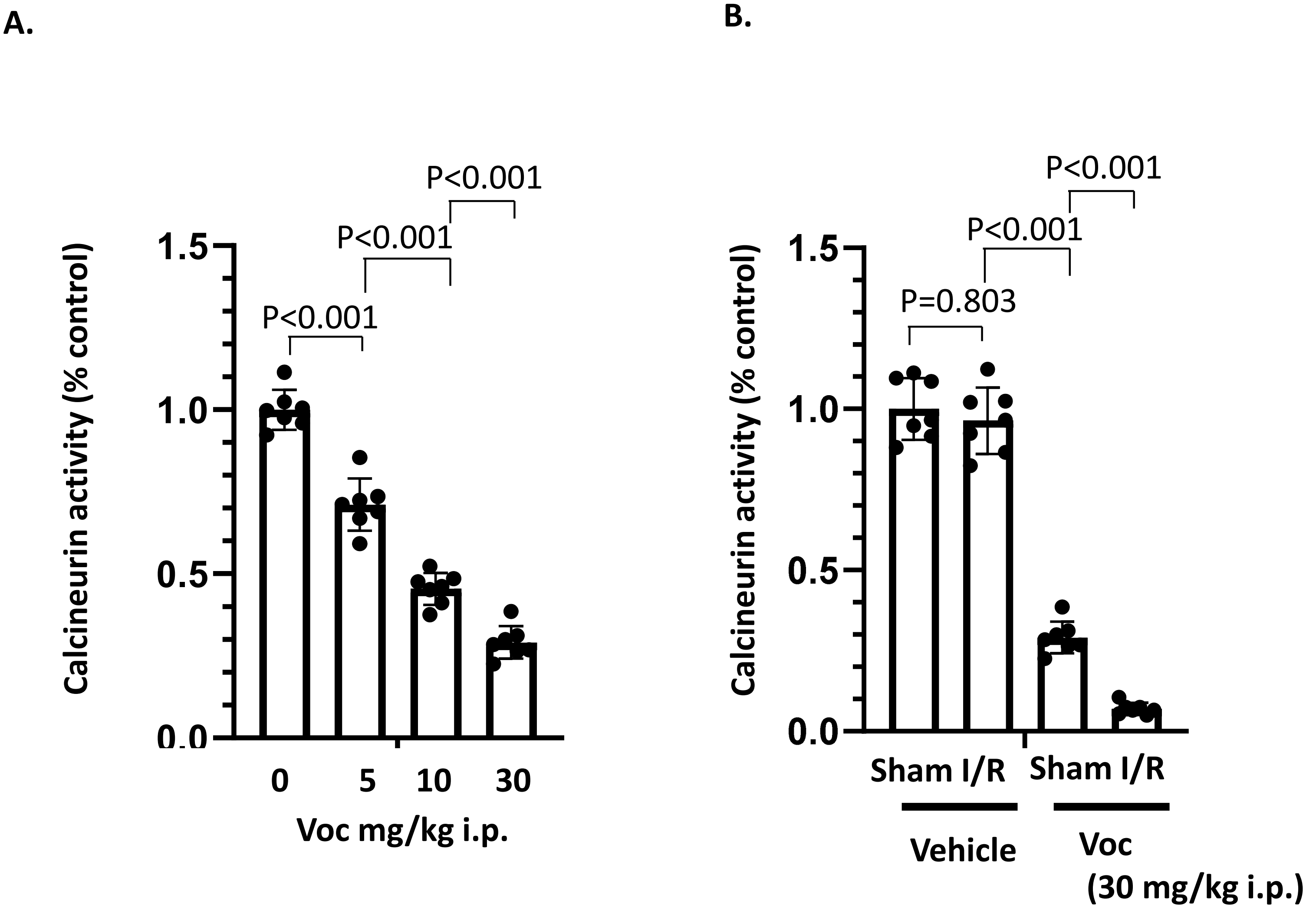

Supplementary Figure 4, Hasegawa et al.

A.

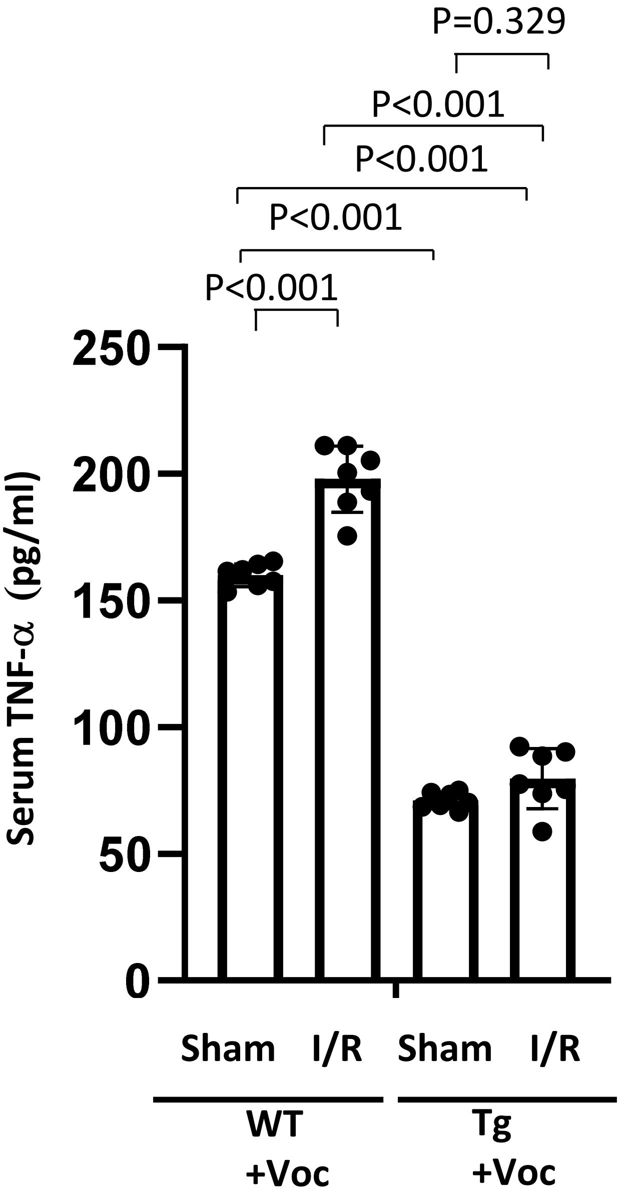

B.

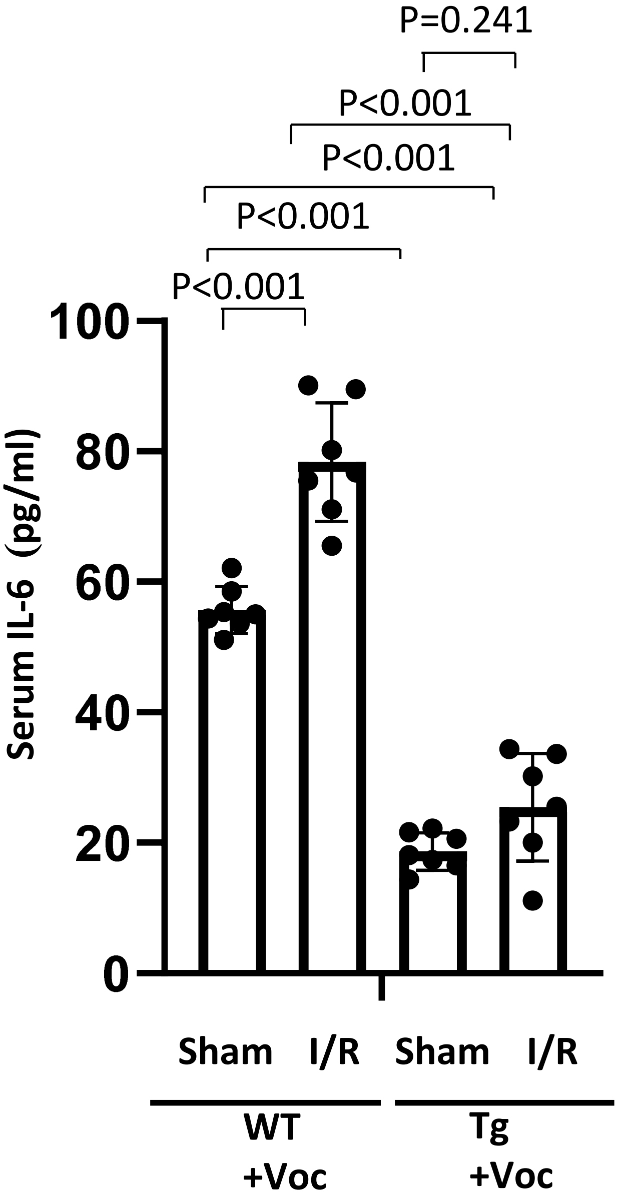

Supplementary Figure 5, Hasegawa et al.

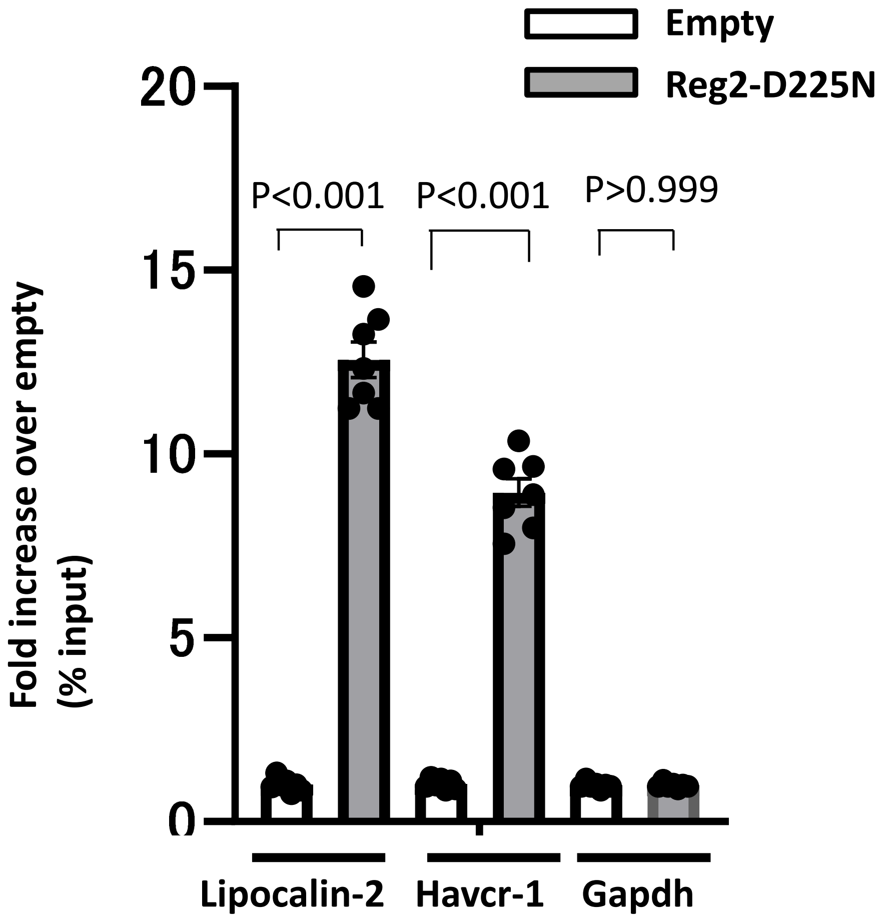

Supplementary Figure 6, Hasegawa et al.

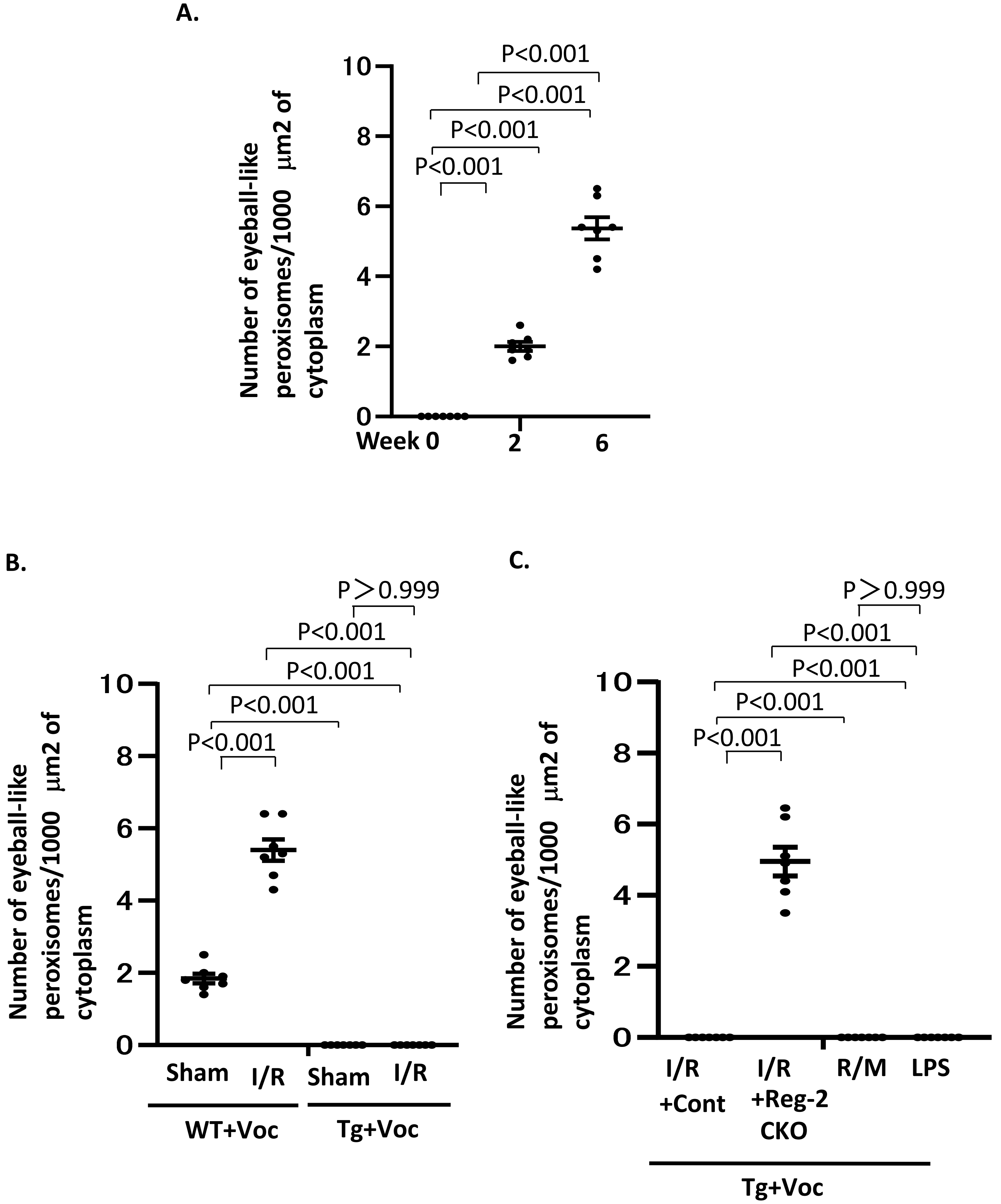

Supplementary Figure 7, Hasegawa et al.

Supplementary Figure Legends

Supplementary Figure 1. Regnase-2 knockdown in proximal tubules induces AKI through accumulation of injury-related transcripts. (A) Real-time PCR analysis of whole kidney tissues from 14-week-old WT mice demonstrated expression of Regnase-1, whereas other canonical RNA decay modulators (TTP, AUF1, TIA1, and UPF) were not found. (B) Comparative analysis of Regnase family members (Regnase-1, -2, -3, and -4) in WT+Voc and Inmt Tg+Voc kidneys at 14 weeks revealed abundant expression of Regnase-1 and Regnase-2, with Regnase-2 markedly upregulated in Inmt Tg+Voc mice. (C) To investigate the renal function of Regnase-2, AAV9 vectors carrying Regnase-2 shRNA were administered via tail vein injection at 11 weeks, and kidney tissues were analyzed at 14 weeks. (D) Immunofluorescence staining confirmed proximal tubular-specific knockdown of Regnase-2 in conditional knockout (CKO) mice, as demonstrated by dual labeling with lotus tetragonolobus lectin (LTL) and quantification of fluorescence intensity. (E) Plasma blood urea nitrogen (BUN) and creatinine (Cr) levels, (F) histological injury scores by hematoxylin and eosin (HE) staining, and (G) urinary albumin excretion all indicated AKI in CKO mice. (H) Bulk RNA sequencing of whole kidneys revealed broad upregulation of injury-related transcripts. Kyoto Encyclopedia of Genes and Genomes (KEGG) enrichment analysis of upregulated differentially expressed genes is demonstrated (x-axis: gene ratio; y-axis: KEGG terms). Circle size represents gene count, and circle color represents adjusted P-value. (I) KEGG pathway analysis emphasized the IL-17 signaling pathway, with Lipocalin-2 (LCN-2) showing prominent induction. (J) Immunostaining for NGAL confirmed significantly increased expression in CKO kidneys, with quantitative data shown to the right. Data were analyzed using two-tailed Student's t-test. Scale bars: 100 μm (immunofluorescence), 50 μm (light microscopy). All quantitative data were obtained from n = 7 mice per group and analyzed using two-tailed unpaired Student's t-tests. Exact P-values are indicated in the figure.

Supplementary Figure 2. Regnase-2 deficiency in proximal tubules induces AKI through mitochondrial and peroxisomal dysfunction. (A) Lipocalin-2 (NGAL), which is upregulated upon Regnase-2 loss, has been reported to interfere with mitochondrial function. Because mitochondria and peroxisomes are functionally interconnected, mitochondrial dysfunction can directly precipitate peroxisomal impairment; therefore, these pathways were examined in detail. (B) Immunofluorescence staining for medium-chain acyl-CoA dehydrogenase, a key enzyme in fatty acid oxidation, revealed a significant reduction in CKO kidneys. Dual labeling with lotus tetragonolobus lectin (LTL) confirmed proximal tubular localization. (C) Expression of the mitochondrial biogenesis regulator PGC-1α and (D) peroxisomal markers, including catalase and acyl-CoA oxidase 1 (ACOX1) as functional indicators, together with PMP70 as a marker of peroxisome abundance, were assessed by real-time PCR of kidney tissue. All were significantly decreased in CKO mice. (E) A summary table illustrates the dynamics of mitochondrial and peroxisomal markers related to their number and function. (F) Renal concentrations of 4-hydroxynonenal (4-HNE) were quantified by ELISA. (G) Apoptosis was evaluated by TUNEL staining. (H) Schematic representation: loss of Regnase-2 leads to increased NGAL, which provokes mitochondrial dysfunction. Elevated oxidative stress, evidenced by increased 4-HNE, triggers peroxisomal impairment, culminating in AKI. (I) An integrated schema summarizes the molecular alterations observed in mitochondria and peroxisomes under Regnase-2 deficiency. All quantitative data were obtained from n = 7 mice per group and analyzed using two-tailed unpaired Student's t-tests. Exact P-values are indicated in the figure. Scale bar: 100 μm.

Supplementary Figure 3. Inmt transgenic mice alone do not ameliorate ischemia/reperfusion-induced AKI. As previously demonstrated, high-dose voclosporin in combination with Inmt transgenic (Tg) mice effectively suppressed AKI induced by ischemia/reperfusion (I/R), lipopolysaccharide (LPS), and rhabdomyolysis (R/M). This protective effect was mediated through Regnase-2 activation, resulting in reduced NGAL expression and, likely, decreased KIM-1 expression via

Havcr-1, thereby preventing mitochondrial and peroxisomal dysfunction. Importantly, this renoprotective effect was attributable to high-dose voclosporin, as Inmt Tg alone did not confer protection against AKI. Quantitative injury scores based on hematoxylin and eosin (HE) staining are displayed for four groups: WT+Sal, WT+I/R, Tg+Sal, and Tg+I/R. All mice were 14 weeks of age. All quantitative data were obtained from n = 7 mice per group and analyzed using one-way ANOVA followed by Bonferroni correction. Exact P-values are indicated in the figure. Scale bar: 50 μm.

Supplementary Figure 4. Dose-dependent inhibition of renal calcineurin activity by voclosporin and enhanced functional drug exposure in ischemic kidneys. (A) Calcineurin (CN) phosphatase activity in kidney cortex homogenates from mice treated with vehicle or voclosporin at 5, 10, or 30 mg/kg intraperitoneally. CN activity is expressed as a percentage of vehicle control. Data are presented as mean ± SEM with individual values. (B) Renal CN phosphatase activity in Sham and ischemia–reperfusion (I/R) kidneys treated with vehicle or voclosporin (30 mg/kg i.p.). CN activity is expressed as a percentage of vehicle-treated Sham controls. CN activity did not differ between Sham and I/R kidneys in vehicle-treated mice ($P = 0.803$). Voc treatment markedly reduced CN activity in Sham kidneys compared with vehicle controls ($P < 0.001$). Importantly, CN activity was further and significantly reduced in the I/R + Voc group compared with the Sham + Voc group ($P < 0.001$), indicating enhanced functional voclosporin exposure in ischemic kidneys. All quantitative data were obtained from n = 7 mice per group and analyzed using one-way ANOVA followed by post-hoc testing. Exact P-values are indicated in the figure.

Supplementary Figure 5. Serum concentrations of TNF-α and IL-6 in wild-type (WT) and INMT-transgenic (Tg) mice subjected to Sham or ischemia–reperfusion (I/R) surgery under voclosporin (Voc) treatment. Cytokine levels were quantified by ELISA. In WT mice, I/R markedly increased serum TNF-α and IL-6 compared with Sham controls (both $P < 0.001$), indicating a robust systemic inflammatory response. In contrast, INMT-Tg mice exhibited significantly lower cytokine levels

after I/R, and the differences between Sham and I/R were not statistically significant (TNF-α: $P = 0.329$; IL-6: $P = 0.241$). These findings demonstrate that INMT overexpression attenuates the I/R-induced systemic inflammatory response despite identical Voc exposure. Data are presented as mean ± SEM with individual values. All quantitative data were obtained from n = 7 mice per group and analyzed using one-way ANOVA followed by post-hoc testing. Exact P-values are indicated in the figure.

Supplementary Figure 6. Regnase-2 directly binds Lcn2 and Havcr1 mRNAs in HEK293 cells. HEK293 cells were transfected with 3×FLAG-Regnase-2 (RNase-dead mutant D225N) or empty vector. RNA–protein complexes were immunoprecipitated using an anti-FLAG antibody, and co-precipitated RNAs were quantified by qPCR. Lcn2 and Havcr1 mRNAs were markedly enriched in FLAG-Regnase-2(D225N) immunoprecipitates, whereas GAPDH mRNA showed no enrichment, confirming specificity. These findings demonstrate direct association of Regnase-2 with Lcn2 and Havcr1 transcripts. All quantitative data were obtained from n = 7 independent experiments and analyzed using two-tailed unpaired Student's t-tests. Exact P-values are indicated in the figure.

Supplementary Figure 7. Quantification of eyeball-like peroxisomes across experimental conditions. (A) The number of eyeball-like peroxisomes per 10^3 μm^2 of cytoplasm was quantified by TEM-based stereology at Weeks 0, 2, and 6 in the high-dose voclosporin model (corresponding to Fig. 1). (B) The density of eyeball-like peroxisomes was calculated as the number of dense-core peroxisomes per 10^3 μm^2 of cytoplasm from seven systematically sampled TEM micrographs per group (corresponding to Fig. 3). I/R+WT+Voc mice exhibited a significant increase in dense-core peroxisomes, whereas both Sham+Tg+Voc and I/R+Tg+Voc mice showed almost no detectable structures. (C) The density of eyeball-like peroxisomes was quantified as in (B) (corresponding to Fig. 6). I/R+Reg-2 CKO mice exhibited a marked increase in dense-core peroxisomes, whereas R/M and LPS models showed almost no detectable structures. Data are presented as mean ± SEM. All quantitative data were obtained from n = 7

mice per group and analyzed using one-way ANOVA followed by Bonferroni correction. Exact P-values are indicated in the figure.

Supplementary Results

Renal calcineurin activity decreases in a dose-dependent manner following intraperitoneal voclosporin administration

To determine the pharmacodynamic effect of voclosporin (Voc) across clinically relevant and supratherapeutic doses, we measured renal calcineurin (CN) phosphatase activity in mice treated with 0, 5, 10, or 30 mg/kg i.p. Voc. CN activity progressively decreased in a clear dose-dependent manner. Compared with vehicle-treated controls, CN activity was significantly reduced at 5 mg/kg ($P < 0.001$), further suppressed at 10 mg/kg ($P < 0.001$), and reached near-maximal inhibition at 30 mg/kg ($P < 0.001$). These results demonstrate that 5–10 mg/kg i.p. Voc produces partial CN inhibition consistent with clinically relevant exposure, whereas 30 mg/kg i.p. induces profound CN suppression, supporting its designation as a high-dose regimen (Fig. S4A).

Ischemia–reperfusion further enhances voclosporin-mediated inhibition of renal calcineurin activity

To determine whether ischemia–reperfusion (I/R) alters the effective renal exposure to voclosporin (Voc), we quantified calcineurin (CN) phosphatase activity in kidney cortex from four groups: Sham + Vehicle, I/R + Vehicle, Sham + Voc (30 mg/kg i.p.), and I/R + Voc (30 mg/kg i.p.). CN activity in vehicle-treated mice did not differ between Sham and I/R kidneys ($P = 0.803$), indicating that I/R alone did not significantly alter basal CN activity. As expected, administration of Voc markedly reduced CN activity in Sham kidneys compared with vehicle controls ($P < 0.001$), confirming the pharmacological effect of Voc (Fig. S4B).

Importantly, CN activity was further and significantly reduced in the I/R + Voc group compared with the Sham + Voc group ($P < 0.001$), despite identical dosing. This additional suppression of CN activity indicates that I/R increases the functional renal exposure to Voc, consistent with enhanced accumulation or reduced clearance of the drug in ischemic kidneys. These findings provide pharmacodynamic evidence

supporting the hypothesis that I/R augments Voc-mediated calcineurin inhibition at the tissue level (Fig. S4B).

INMT overexpression attenuates the systemic inflammatory response induced by I/R under voclosporin treatment

To evaluate whether the Voc/INMT axis modulates the systemic inflammatory response after I/R injury, we measured serum TNF-α (Fig. S5A) and IL-6 levels (Fig. S5B) in WT and INMT-transgenic (Tg) mice subjected to Sham or I/R surgery under voclosporin treatment. In WT mice, I/R markedly increased serum TNF-α and IL-6 compared with Sham controls (both $P < 0.001$), indicating a robust systemic inflammatory response. In contrast, INMT-Tg mice exhibited significantly lower cytokine levels after I/R, and the differences between Sham and I/R were not statistically significant (TNF-α: $P = 0.329$; IL-6: $P = 0.241$). These findings demonstrate that INMT overexpression suppresses the I/R-induced systemic cytokine response despite identical Voc exposure, suggesting that the Voc/INMT axis attenuates systemic inflammation in addition to its renal effects.

Regnase-2 directly associates with Lcn2 and Havcr1 mRNAs

To determine whether Regnase-2 physically interacts with its putative target transcripts, we performed RIP–qPCR using HEK293 cells expressing 3×FLAG-tagged Regnase-2(D225N), an RNase-inactive mutant that preserves RNA-binding capacity. Immunoprecipitation of FLAG-Regnase-2(D225N) resulted in a robust enrichment of Lcn2 and Havcr1 mRNAs compared with empty-vector controls. In contrast, GAPDH mRNA, which lacks Regnase-responsive stem-loop motifs, showed no detectable enrichment. These results demonstrate that Regnase-2 directly binds Lcn2 and Havcr1 transcripts in cells, independent of its RNase activity (Fig. S6).

Quantification of Eyeball-Like Peroxisomes During High-Dose Voclosporin Treatment (Related to Fig. 1)

Quantitative TEM stereology demonstrated a clear time-dependent increase in eyeball-like peroxisomes during high-dose voclosporin (Voc) treatment. The number of eyeball-like peroxisomes per 10^3 μm^2 of cytoplasm was almost undetectable at week 0, modestly increased at week 2, and markedly elevated at week 6 (all comparisons $P < 0.001$), consistent with the ultrastructural changes observed in electron micrographs (Fig. S7A).

Genotype-Dependent Formation of Eyeball-Like Peroxisomes Under I/R Stress (Related to Fig. 3)

Quantification of eyeball-like peroxisomes revealed a marked genotype-dependent difference under ischemia/reperfusion (I/R) stress. Using TEM-based stereology, the number of eyeball-like peroxisomes per 10^3 μm^2 of cytoplasm was significantly increased in the I/R+WT+Voc group, whereas both Sham+Tg+Voc and I/R+Tg+Voc groups showed almost no detectable dense-core peroxisomes ($P < 0.001$ vs WT). Notably, the I/R+Tg+Voc group remained indistinguishable from its sham counterpart ($P > 0.999$), indicating that Inmt overexpression completely prevented the formation of dense-core peroxisomes under high-dose Voc and I/R (Fig. S7B) .

Reg-2 Deficiency Exacerbates Eyeball-Like Peroxisome Formation Under I/R Stress (Related to Fig. 6)

Reg-2 deficiency markedly increased the formation of eyeball-like peroxisomes under I/R stress.

Using TEM-based stereology, the number of eyeball-like peroxisomes per 10^3 μm^2 of cytoplasm was dramatically elevated in the I/R+Reg-2 CKO group, whereas the I/R+control group showed only a modest increase. In contrast, neither the Rhabdomyolysis (R/M) nor LPS models exhibited detectable dense-core peroxisomes. These findings indicate that dense-core peroxisome formation is a highly specific ultrastructural response to I/R stress under Reg-2 deficiency (Fig. S7C).

Supplementary Discussions

In this study, we sought to clarify the pharmacological range of voclosporin (Voc) in the context of renal ischemia–reperfusion (I/R) injury and to determine whether I/R alters the effective renal exposure to Voc. To address this, we combined a dose–response analysis with an I/R experiment, enabling a comprehensive evaluation of both the magnitude of calcineurin (CN) inhibition and the influence of ischemic injury on Voc pharmacodynamics.

Our dose–response experiment demonstrated a clear graded suppression of renal CN activity across 5, 10, and 30 mg/kg i.p. Voc. The 5 mg/kg dose, which corresponds to the human clinical exposure after body surface area conversion and adjustment for oral bioavailability, produced only mild CN inhibition. The 10 mg/kg dose, widely used in previous murine inflammatory models, resulted in moderate CN suppression. In contrast, 30 mg/kg i.p. induced near-maximal CN inhibition, clearly distinguishing it from clinically relevant doses. These findings support the designation of 30 mg/kg i.p. as a pharmacologically supratherapeutic "high-dose" regimen.

We further examined whether I/R modifies the renal handling of Voc. Although I/R alone did not significantly alter basal CN activity, Voc-treated I/R kidneys exhibited a significantly greater reduction in CN activity compared with Voc-treated Sham kidneys, despite identical dosing. Because CN inhibition directly reflects intracellular exposure to calcineurin inhibitors, this additional suppression indicates that I/R enhances the functional renal accumulation or retention of Voc. These pharmacodynamic data provide mechanistic support for our hypothesis that ischemic injury increases Voc exposure within the kidney, which may contribute to the exacerbation of nephrotoxicity under I/R conditions.

Limitations

A key limitation of this study is that direct quantification of Voc concentrations in blood or kidney tissue could not be performed. LC-MS/MS measurement of Voc requires proprietary analytical standards and validated internal controls that are not commercially available and remain restricted to pharmaceutical

development laboratories. As a result, establishing a de novo LC-MS/MS assay is not technically feasible in an academic setting. To overcome this limitation, we employed renal CN activity as a pharmacodynamic surrogate for tissue Voc exposure. Although CN activity provides a biologically meaningful and widely accepted functional readout of calcineurin inhibitor exposure, it does not allow direct determination of absolute Voc concentrations. Future studies incorporating validated LC-MS/MS assays, once accessible, will be important to confirm the pharmacokinetic basis of the enhanced CN inhibition observed under I/R conditions.

Together, the dose–response and I/R experiments provide a coherent framework for interpreting the effects of Voc in our model. The data demonstrate that 30 mg/kg i.p. represents a high-dose regimen relative to both previously reported murine studies and human clinical dosing, and that I/R further augments the effective renal exposure to Voc. These findings highlight the importance of considering both dose selection and renal perfusion status when evaluating the nephrotoxic potential of calcineurin inhibitors.

Although our experimental strategy relies on proximal tubule–specific overexpression of Inmt, direct transgenic augmentation is not currently feasible in clinical practice. Nevertheless, several emerging molecular pathways suggest potential avenues for pharmacological INMT enhancement.

First, sigma non-opioid intracellular receptor 1 (SIGMAR1) has been identified as a receptor for INMT-derived tryptophan metabolites[48], and INMT itself is considered an endogenous SIGMAR1 agonist. Activation of SIGMAR1 may therefore potentiate downstream INMT-related cytoprotective pathways, raising the possibility that SIGMAR1-targeting compounds could indirectly augment INMT activity.

Second, recent studies have identified the lysine methyltransferase SMYD3 as an epigenetic regulator of INMT transcription[49]. Pharmacological modulation of SMYD3 or related chromatin-regulatory mechanisms may represent a feasible strategy to increase endogenous INMT expression.

Although the roles of SIGMAR1 and SMYD3 in renal tissues remain largely unexplored, these pathways provide promising directions for developing INMT-augmenting therapies. Future studies investigating these regulatory mechanisms will be essential for translating INMT-mediated renal protection into clinically applicable interventions, as the reviewer suggested.

The dual effects of high-dose Voc can be understood as an INMT-dependent switch in the dominant biological pathway. In WT mice, Voc suppresses INMT and triggers IAA accumulation, leading to rapid tubular injury that masks any protective signaling. In contrast, when INMT is preserved, IAA does not accumulate, and Voc no longer induces nephrotoxicity. Under this protected metabolic state, high-dose Voc robustly induces Reg-2, which suppresses Lcn2 and Havcr1 expression and attenuates tubular injury. A major limitation is that the mechanism of Reg-2 induction cannot be directly determined: high-dose Voc alone causes AKI too rapidly to study upstream events, whereas high-dose Voc＋Inmt Tg alters tryptophan/indole metabolism, complicating interpretation. This structural constraint of the model prevents direct identification of the Reg-2 activation mechanism.

Our TEM-based stereological analysis revealed a distinctive pattern of peroxisomal remodeling characterized by the formation of dense-core (“eyeball-like”) peroxisomes. This phenotype increased progressively during high-dose Voc treatment, was markedly induced by I/R under Voc exposure, and was dramatically exacerbated by Reg-2 deficiency. In contrast, R/M and LPS models showed almost no dense-core peroxisomes, indicating that this structure represents a specific ultrastructural signature of I/R stress in the context of impaired INMT–Reg-2 signaling.

Because dense-core peroxisomes cannot be detected by immunofluorescence, TEM stereology provides the most accurate method for evaluating this phenotype. The binary nature of the dense-core structure ensures objective classification, and the consistent findings across Figures 1, 3, and 6 highlight the robustness of this morphological marker.

These results support a model in which Reg-2 preserves peroxisomal integrity, preventing the formation of dense-core peroxisomes and maintaining mitochondrial–peroxisomal homeostasis. Loss of Reg-2 leads to peroxisomal dysfunction, ROS accumulation, and tubular injury, establishing Reg-2 as a key regulator of peroxisomal stress responses.

In addition to its renal effects, our data indicate that the Voc/INMT axis also modulates the systemic inflammatory response induced by I/R injury. I/R markedly increased circulating TNF-α and IL-6 levels in WT mice, consistent with the well-established systemic inflammatory response syndrome triggered by ischemic injury. However, INMT-Tg mice exhibited significantly attenuated cytokine responses despite receiving the same Voc dose. Because Regnase-2 is known to degrade mRNAs encoding IL-6, TNF-α, and other inflammatory mediators, these findings suggest that INMT-mediated stabilization of Regnase-2 contributes to the suppression of systemic inflammation. This systemic anti-inflammatory effect may have important implications for critical care settings, where I/R-induced cytokine storms contribute to multi-organ dysfunction.

Although high-dose Voc exhibited strong renoprotective effects under INMT preservation, this dosing strategy is not currently feasible in clinical practice. The approved clinical dose for lupus nephritis is substantially lower, and Voc lacks therapeutic drug monitoring, making high-dose administration difficult to control safely. In wild-type mice, high-dose Voc rapidly induces AKI, and its protective effect becomes evident only when INMT expression is preserved, preventing IAA accumulation and allowing Reg-2 activation. Therefore, the present findings should be interpreted as revealing a biological mechanism rather than proposing immediate clinical use of high-dose Voc.

The translational implication of this study lies in identifying the INMT–Reg-2 axis as a potential therapeutic target. Future approaches may include pharmacological enhancement of INMT expression, development of TDM systems enabling safe modulation of Voc exposure, or the design of new agents

capable of activating Reg-2 without inducing nephrotoxicity. These strategies may ultimately allow the protective mechanism uncovered here to be leveraged in clinical settings.

Across all models, renal function assessed by BUN and creatinine closely paralleled the molecular and ultrastructural findings. Inmt Tg mice maintained normal BUN and creatinine levels despite Voc＋I/R stress, whereas Reg-2 CKO mice exhibited marked renal dysfunction. These results highlight the functional relevance of the Inmt–Reg-2 axis, demonstrating that preservation of peroxisomal and mitochondrial integrity translates directly into protection of renal function. Accurate GFR measurement in mice requires invasive clearance assays that are difficult to perform in AKI models, and non-invasive FITC-sinistrin sensors were not available in our facility. Therefore、BUN and creatinine were used as standard and reliable renal functional indices, which consistently supported the mechanistic conclusions of this study.

Supplementary Methods

AAV9 injection

Adeno-associated virus serotype 9 (AAV9) vectors containing short hairpin RNA (shRNA) and targeting mouse Regnase-2 and empty control vectors were constructed and purchased from Takara Bio (Kusatsu, Shiga, Japan). The AAV9 titer after packaging was 8.5 × 10^{13} vector genomes/mL, and each mouse received a dose of 5 × 10^{12} vector genomes, corresponding to an injection volume of approximately 60 μL. At 11 weeks of age, mice were administered AAV9 carrying Regnase-2 shRNA via tail vein injection to suppress Regnase-2 expression. Mice receiving AAV9 with empty vectors were used as controls. Kidney tissues were harvested 5 weeks post-injection (16 weeks of age), and knockdown efficiency was assessed by immunofluorescence. Consistent with previous reports, AAV9 preferentially transduced renal proximal tubules.[50] The shRNA oligonucleotide sequences were as follows:

· Control shRNA: 5′-UUCUCCGAACGUGUCACGUTT-3′, 3′-ACGUGACACGUUCGGAGAATT-5′

· Regnase-2 shRNA: 5′-ACCUUAGAGCUUCUCUUGAUATT-3′, 3′-TTAUCAAGAGAAGCUCUAAGGU-5′

Plasmid construction.

Regnase-2 cDNA was inserted into a pFlag-CMV2 vector (Sigma-Aldrich), as previously described.[51] Full-length (1–400) or partial fragments (1–300, 400–500) of the Lipocalin-2 3′ -UTR sequence were cloned into a pGL3 vector (Promega). The 3′ -UTR cDNA of β-globin (1–130), with or without the Lipocalin-2 3′ -UTR fragment (320–370), as well as the 3′ -UTR cDNAs of Havcr-1 (1–965), Ccl2 (1–469), and Cxcl1 (1–591), were also placed into the pGL3 vector. Lipocalin-2 CDS and Lipocalin-2 CDS plus 3′ -UTR were cloned into the pTREtight vector (Clontech). Importantly, the expression efficiency

of Regnase-2 cDNA from the pFlag-CMV2 vector was verified in preliminary experiments, ensuring the reliability of subsequent overexpression studies.

Stability of mRNA in proximal tubular cells

Proximal tubules (1×10^6 cells) harvested from wild-type and Regnase-2 CKO mice were used. Actinomycin D (2 μg/mL) was added to the culture medium to block transcription, and total RNA was extracted at the indicated time points. Transcript levels of Lipocalin-2, Havcr-1, Ccl2, Cxcl1, and Gapdh were quantified by quantitative real-time PCR (qPCR). Relative expression was normalized to Gapdh, and mRNA stability was assessed by comparing decay kinetics between wild-type and CKO cells.

Tet-off system

HEK293 Tet-off cells (3×10^6) were transfected with either pTREtight-Lipocalin-2-CDS or pTREtight-Lipocalin-2-CDS + 3′-UTR, together with Regnase-2 expression plasmids or an empty control vector. After 3 h, the cells were subdivided into three 60-mm dishes and cultured overnight. Transcription from pTREtight vectors was terminated by the addition of doxycycline (1 μg/mL), and total RNA was extracted at the indicated time points. Lipocalin-2 and Gapdh mRNA levels were measured by quantitative real-time PCR (qPCR). Relative expression was normalized to Gapdh, and transcript stability was evaluated based on time-dependent changes in mRNA abundance.

Luciferase assay

HEK293 cells were transfested with pGL3-Lipocalin-2 3′-UTR plasmids or pGL3-empty plasmid together with Regnase-2 expression plasmid or empty control plasmid. After 48 h of cultivation, cells were lysed, and luciferase activities in the resulting lysates were detected using the Dual-Luciferase Reporter Assay System (Promega). The Renilla luciferase gene was simultaneously transfected as an internal control.

RNA fold

The minimum-free energy (MFE) secondary structure of the entire mRNA construct was predicted using the RNAfold web server,[52] the secondary structure of an RNA sequence that contributes the least free energy is a MFE structure. A loop-based energy model and the dynamic programming approach were applied to forecast this structure.[53] An RNA secondary structure can be uniquely divided into loops and external bases.

RNA isolation, reverse transcription, and PCR.

RNA processing, isolation, and PCR detection were performed using previously established protocols with the same instruments and reagents, as outlined in our earlier studies.[54-56] Primers were selected to meet the specific experimental requirements of the present study, and their sequences are listed in Table 1. These procedures have been consistently used in prior publications from our group, ensuring methodological reliability and reproducibility.

Voclosporin administration.

Voclosporin (30 mg/kg) was administered via an intraperitoneal injection daily for 2 weeks (n = 8/group) from 8 to 10 weeks of age, preceded by a 3-day preparatory period, during which solvent (3% ethanol in sunflower oil) was administered intraperitoneally to acclimate the animals to the treatment. The control group received only the solvent daily. To mitigate cage-dependent effects, the treatments were administered in mixed cages. The dose of voclosporin was selected, as previously described by our group.[55]

Transgenic mice

Inmt transgenic mice (Inmt Tg), which overexpress Inmt specifically in proximal tubules, were generated according to the existing protocols.[2] Control littermates were used for the comparative analysis. The Inmt Tg line was originally established by our group and has been reported in JASN, where it was shown to prevent voclosporin overdose-induced peroxisomal structural changes and AKI through renal indole detoxification.[2] This well-established model, developed, and characterized in our laboratory, provides a platform for mechanistic studies of Regnase-2–mediated pathways in AKI in this study.

RNA sequencing

RNA sequencing was performed to compare gene expression profiles in kidneys harvested from 14-week-old mice

(1) High-dose voclosporin–treated Inmt transgenic (Tg) mice subjected to ischemia–reperfusion (I/R) injury were compared with high-dose voclosporin–treated wild-type (WT) mice subjected to I/R.

(2) Regnase-2 conditional knockout (CKO) mice generated by administration of AAV9 vectors carrying shRNA against Regnase-2 were compared with mice receiving control shRNA.

RNA was extracted from a left kidney tissue, following the protocol used for quantitative polymerase chain reaction (PCR). Libraries were prepared using the TruSeq Stranded mRNA kit (Illumina, San Diego, CA, USA), and sequencing was carried out on a NovaSeq platform (Illumina), yielding an average read length of 100 bp.

Sequence alignment was conducted, using STAR (version 2.7.3a), raw read counts were obtained with HTSeq, and normalization was done with DESeq2. Gene expression levels were estimated with Cufflinks (version 2.2.1) and reported as fragments per kilobase of transcript per million mapped reads (FPKM). Data quality was assessed with FastQC, and low-quality reads were excluded from further

analysis. Overrepresentation, gene set enrichment, and pathway analyses were performed to identify significant differences in protein-coding gene expression.

The accession number for the raw and processed sequencing data will be provided in the Gene Expression Omnibus upon acceptance of the manuscript or upon notification of acceptance in principle.

Serum metabolite quantification

Serum indole-3-acetic acid (IAA) levels were quantified using a commercial ELISA kit (abx150354; Abbexa, Cambridge, UK) according to the manufacturer's instructions and our previously reported experience.[2] Serum creatinine and blood urea nitrogen (BUN) levels were quantified (mg/dL), using an automated chemistry analyzer. Albuminuria was assessed by sodium dodecyl sulfate–polyacrylamide gel electrophoresis (SDS-PAGE).

Electron microscopy

After mouse kidney specimens had been immersed in Epon epoxy resin (Hexion, Columbus, OH., USA), electron micrographs of 10 proximal tubular cells per kidney were randomly selected for morphometric evaluation.

Immunofluorescence staining

Dual-labeling immunofluorescence staining was performed on 5-µm cryostat kidney sections, following overnight incubation with validated primary and secondary antibodies. The primary antibodies included: rabbit polyclonal anti-Inmt (Thermo Fisher Scientific, Waltham, MA, USA; Cat# PA5-25390), rabbit monoclonal anti-medium-chain acyl-CoA dehydrogenase (MCAD) (Abcam, Cambridge, UK; Cat# ab92461), mouse monoclonal anti-aquaporin-1 (AQP-1; clone B-11) (Santa Cruz Biotechnology, Dallas, TX, USA; Cat# sc-25287), rabbit polyclonal anti-Regnase-2 (Proteintech Group, Rosemont, IL, USA;

Cat# 26773-1-AP), and biotinylated lotus tetragonolobus lectin (LTL) (Vector Laboratories, Burlingame, CA, USA; Cat# L-132). Secondary antibodies were obtained from Jackson ImmunoResearch Laboratories (West Grove, PA, USA). Double immunofluorescence staining for TUNEL and AQP-1 was also done to localize apoptosis provoked by voclosporin treatment or Inmt deficiency.

All antibodies (Inmt, MCAD, AQP-1, and LTL) have been extensively validated in our laboratory and were previously employed in our group,[2] ensuring high sensitivity and specificity based on direct experimental experience and peer-reviewed publication. For Regnase-2, immunofluorescence staining was newly incorporated in the present study, guided by and consistent with two independent reports that had established the reliability of this antibody in murine tissues.[57,58] Thus, all staining procedures were performed with rigorously validated antibodies, ensuring methodological accuracy and reproducibility.

Immunostaining.

Kidney specimens for histology were prepared, and 4-μm paraffin sections were obtained after fixation. The latter were stained with primary antibodies against albumin (Nordic-MUbio, Susteren, Netherlands) and neutrophil gelatinase-associated lipocalin (NGAL; Abcam, Cambridge, MA, USA; Cat# ab70287). Labeling with biotin-conjugated goat anti-rabbit immunoglobulin G was followed by treatment with the Vectastain Elite ABC Kit (Vector Laboratories, Newark, CA, USA), and final imaging was carried out using a 3CCD camera. NGAL immunostaining was conducted using the same protocol previously established and reported by our group,[2] ensuring methodological continuity.

Enzyme-linked immunosorbent assay (ELISA).

ELISA kits were used to quantify 4-hydroxynonenal (4-HNE) in cell lysates prepared from murine kidneys. 4-HNE levels were measured using a commercial assay kit (Abcam, Cambridge, UK; Cat# ab238538). Samples underwent processing according to the manufacturer's instructions, and absorbance was recorded at 450 nm with a microplate reader.

Calcineurin Phosphatase Activity Assay

Renal calcineurin activity assay (I/R experiment and dose–response experiment)

Renal calcineurin (CN) phosphatase activity was quantified to evaluate the pharmacodynamic effect of voclosporin (Voc) under ischemia–reperfusion (I/R) conditions and across different intraperitoneal (i.p.) doses. Two experimental designs were performed:

(1) I/R experiment comparing Sham and I/R kidneys with or without high-dose Voc, and

(2) Dose–response experiment assessing CN inhibition at 0, 5, 10, and 30 mg/kg i.p. Voc.

Tissue collection

Immediately after euthanasia, kidneys were excised, rinsed in ice-cold PBS, and the cortex was dissected and snap-frozen in liquid nitrogen. Samples were stored at −80°C until analysis.

Preparation of kidney homogenates

Frozen cortex tissue was homogenized in ice-cold lysis buffer (50 mM Tris-HCl pH 7.5, 1 mM DTT, 1 mM ascorbate, 0.02% NP-40, protease inhibitor cocktail). Homogenates underwent three freeze–thaw cycles followed by centrifugation at 13,000 × g for 10 min at 4°C. Supernatants were collected for CN activity measurement. Total protein concentration was determined using the BCA assay.

Calcineurin phosphatase activity measurement

CN activity was measured using a commercially available ELISA-based assay (Calcineurin Cellular Activity Assay Kit, Enzo Life Sciences, Farmingdale, NY, USA; Cat. No. BML-AK816) employing the RII phosphopeptide substrate according to the manufacturer's instructions. Briefly, samples were incubated with reaction buffer containing Ca^{2+} and calmodulin, and phosphate release was quantified colorimetrically at 620 nm. CN activity was normalized to total protein and expressed as percentage of vehicle-treated Sham controls.

Experimental groups

I/R experiment:

· Sham + Vehicle

· I/R + Vehicle

· Sham + Voc (30 mg/kg i.p.)

· I/R + Voc (30 mg/kg i.p.)

Dose–response experiment:

· Vehicle (0 mg/kg)

· Voc 5 mg/kg i.p. (clinical-equivalent dose)

· Voc 10 mg/kg i.p. (dose used in prior murine studies)

· Voc 30 mg/kg i.p. (high-dose)

This assay provides a direct functional readout of calcineurin inhibition and therefore reflects the effective renal exposure to voclosporin under both I/R conditions and graded dosing. The combined analysis allows evaluation of (i) whether I/R enhances functional Voc accumulation and (ii) how 30 mg/kg compares with clinically relevant doses.

Transmission electron microscopy and stereological quantification

Kidney cortex samples were fixed, embedded, sectioned, and stained as described above. Ultrathin sections were imaged at fixed magnifications (×12,700 for overview; ×42,200 for high-magnification analysis). For quantitative analysis, seven systematically sampled TEM micrographs per group were obtained using systematic uniform random sampling. Cytoplasmic area was measured using ImageJ, and the density of peroxisomes was expressed as the number per 10^3 μm^2 of cytoplasm.

Definition of eyeball-like peroxisomes:

Structures were classified as eyeball-like peroxisomes only when they exhibited:

1. A sharply demarcated electron-dense core,

2. An intact peroxisomal membrane, and

3. Preserved peroxisomal matrix surrounding the core.

These criteria represent a binary morphological definition, minimizing subjectivity. Classification was performed in a blinded manner. The same stereological method was applied to Figures 1, 3, and 6.

RNA Immunoprecipitation (RIP)–qPCR

RNA immunoprecipitation was performed to assess the direct association between Regnase-2 and target mRNAs. HEK293 cells were seeded in 6-well plates and transfected with expression vectors encoding N-terminal 3×FLAG-tagged mouse Regnase-2 (wild-type or RNase-dead mutant D225N) or empty vector using Lipofectamine 2000 (Thermo Fisher Scientific).

The D225N mutant was selected because Asp225 represents the first catalytic Asp residue within the conserved D–E–D–H tetrad of the PIN-like RNase domain of Regnase-2, corresponding to Asp141 in Regnase-1. Mutation of this residue abolishes Mg^{2+}-dependent endoribonuclease activity in Regnase/MCPIP family proteins, and is therefore widely used as the functionally validated RNase-inactive form.

Twenty-four hours after transfection, cells were washed with ice-cold PBS and lysed in RIP lysis buffer (50 mM Tris-HCl pH 7.4, 150 mM NaCl, 1% NP-40, 1 mM EDTA, 1 mM DTT, protease inhibitor cocktail, and RNase inhibitor) on ice for 20 min. Lysates were cleared by centrifugation at 12,000 × g for 10 min at 4°C, and a fraction of the supernatant was saved as input.

For RIP, cleared lysates were incubated with anti-FLAG M2 antibody (Sigma-Aldrich) pre-bound to Protein G magnetic beads for 3–4 h at 4°C with gentle rotation. Normal mouse IgG was used as a negative control. Beads were washed four times with high-salt wash buffer (50 mM Tris-HCl pH 7.4, 300 mM NaCl, 0.5% NP-40, 1 mM EDTA, RNase inhibitor) and once with PBS. RNA bound to immunoprecipitated complexes was extracted using TRIzol reagent (Thermo Fisher Scientific). Input RNA was isolated in parallel.

Purified RNA was treated with DNase I and reverse-transcribed using random hexamers and oligo(dT) primers. Quantitative PCR was performed to measure Lcn2 and Havcr1 mRNAs. GAPDH mRNA was used as the non-target negative control, as it lacks Regnase-2-responsive stem-loop motifs and is not regulated by Regnase-2. Enrichment was calculated relative to input and IgG controls.

Statistical analysis

All statistical analyses were conducted using Prism 8 (GraphPad Software, San Diego, CA, USA). Sample size was determined based on power calculations to ensure statistical robustness, while adhering to the three reductions (3R) principle. Descriptive data were presented as means ± standard error, and group comparisons were performed.

Supplementary Tables:

Supplementary Table 1: Primers used to amplify the related genes

Name	Forward	Reverse
Inmt	5′-CCTACGACTGGTCCTCCATAG-3′	5′-CTTCTGAGCTTGGCTTCCTT-3′
Lcn2	5′-CAGTGTCGATGTTGGATGCC-3′	5′-AGAACAGACTGGATGCCCTG-3′
Havcr1	5′-CCAAGGACACCTGGGACAAC-3′	5′- AGGCTCTGGTAGGTTGTTGC -3′
Ccl2	5′- TTAAAAACCTGGATCGGAACCAA -3′	5′- GCATTAGCTTCAGATTTACGGGT -3′
Cxcl1	5′- GCTGGGATTCACCTCAAGAAC -3′	5′- TGTGGCTATGACTTCGGTTTGG -3′
Regnase-1	5′- ACTGCTGAGTTCCTGCTTCT -3′	5′-GGTCTTGATGGTGCTGTTGT -3′
Regnase-2	5‘- TCCACCTGCTACATCTTCCTC -3′	5′- GGCTGCTTCTGATGTTCTTGA -3′
Regnase-3	5′- TGGAGGAGCTGTTTGAGGAT -3′	5′- CTGCTTCTGGTTGGTGTTGT -3′
Regnase-4	5′- AGCTGCTGAGGAGTTTGAGA -3′	5′- GCTCTTGCTGGTGTAGTTGC -3′
Zfp36	5′- CAGTGCTGAGTCTGTTTGGA -3′	5′- CACAGTTGCTGGTGATGAGC -3′
Hnrnpd	5′- ATGGAGCAGTTCTACCGTGA -3′	5′-TCTGCTGTTGCTGTTCTTGG -3′
Tia1	5′- CAGCAGATGACCTACCGTCT -3′	5′- GTGCTTCTTCAGCTGCTTCT -3′
UPF1	5′-GGACATCAGCAGTTCCAGAG -3′	5′- CTGCTGATCGTCTTGGTGTC -3′
Ppargc1a	5′- GAGGAGAGAGATGAGGAGGC -3′	5′- CTGTTGGTCTTGGTGGTGTT -3′
CAT	5′- CCTGGTGTTGTTGAGGAGGA -3′	5′- AGGTCTTGATGGTGGTGTTG -3′
Acox1	5′- TGCTGAGGAGTTTGAGGAGA -3′	5′- CTGCTTCTGGTTGGTGTTGT -3′
Abcd3	5′- GCTGCTGAGAGAGATGAGGA -3′	5′- CTGCTGTTGGTCTTGGTGAT -3′
Gapdh	5′-CCAGGGCTGCTTTTAACTC-3′	5′-GCTCCCCCCTGCAAATGA-3′

Inmt, Indolethylamine N-methyltransferase; Havcr-1, Hepatitis A virus cellular receptor 1 (Kidney injury molecule-1, KIM-1); Ccl2, C-C motif chemokine ligand 2 (Monocyte chemoattractant protein-1, MCP-

1); Cxcl1, C-X-C motif chemokine ligand 1; TTP, Tristetraprolin (Zfp36); AUF1, AU-rich element RNA-binding protein 1; TIA-1, T-cell-restricted intracellular antigen-1; Upf1, Regulator of nonsense transcripts 1 (UPF1 RNA helicase and ATPase); PGC1α, Peroxisome proliferator-activated receptor gamma coactivator 1-alpha (Ppargc1a); ACOX1, Acyl-CoA oxidase 1; PMP70, Peroxisomal membrane protein 70 (Abcd3); Gapdh, Glyceraldehyde-3-phosphate dehydrogenase.

Supplementary Table 2. Clinical data of patients with acute kidney injury as confirmed by needle kidney biopsy

Sample	All patients (N =10)	CNI-induced (n = 5)	Non-CNI (n = 5)
**Age, years**	63 ± 15	62 ± 20	63 ± 13
**Female ratio, n (%)**	0	0	0
**eGFR (mL/min/1.73 m^2)**	40 ± 17	39 ± 16	40 ± 19
**Proteinuria (g/d)**	6.1 ± 0.2	6.3 ± 0.2	6.0 ± 0.2

Values are expressed as mean ± standard deviation. eGFR, estimated glomerular filtration rate; AKI, acute kidney injury; CNI, calcineurin inhibitor.

Supplementary Table 3. Genes upregulated in kidneys of *Inmt* transgenic mice+high dose Voc-induced acute kidney injury (TG AKI) compared with wild-type mice+high dose Voc-induced AKI (WT AKI)

Transcript_ID	Gene_Symbol	Description	TGAKI/WTAKI.fc	TGAKI/WTAKI.raw.pval
NM_010235	Fosl1	fos-like antigen 1	-1228.463143	1.21583E-13
NM_023256	Krt20	keratin 20	-1091.792101	6.06318E-16
NM_001166631	Havcr1	hepatitis A virus cellular receptor 1	-420.230963	6.11162E-14
NM_001270511	Gm10352	predicted gene 10352	-346.434194	2.98403E-10
NM_001270510	Gm10256	predicted gene 10256	-342.210592	3.20208E-10
NM_001166384	Rbmy	RNA binding motif protein, Y chromosome	-326.020117	4.28078E-10
NM_001357493	Saa1	serum amyloid A 1	-245.950815	1.36552E-10
NM_001270514	Gm21693	predicted gene, 21693	-201.102407	6.59945E-10
NM_001270512	Gm3376	predicted gene 3376	-194.384511	9.86308E-09
NM_001270515	Gm21704	predicted gene, 21704	-169.260165	1.88188E-09
NR_003548	Sprr2g	small proline-rich protein 2G	-155.522213	2.25984E-09
NM_001039701	Il1rn	interleukin 1 receptor antagonist	-145.238825	1.37826E-09
NM_001270513	Gm21677	predicted gene, 21677	-143.342061	5.15504E-09
NM_001270516	Gm21708	predicted gene, 21708	-137.417923	6.63195E-09
NM_008491	Lcn2	lipocalin 2	-133.172602	8.2833E-11
NM_009264	Sprr1a	small proline-rich protein 1A	-104.259489	4.70307E-10
NM_008176	Cxcl1	chemokine (C-X-C motif) ligand 1	-80.491072	4.11054E-09
NM_001270518	Gm4064	predicted gene 4064	-78.939382	1.91082E-06
NM_008871	Serpine1	serine (or cysteine) peptidase inhibitor, clade E, member 1	-78.257134	2.4985E-09
NM_001291066	Adam8	a disintegrin and metallopeptidase domain 8	-72.569148	1.34814E-08
NM_001081185	Flnc	filamin C, gamma	-70.179308	6.3051E-09
NM_019450	Il1f6	interleukin 1 family, member 6	-62.079326	5.32259E-07
NM_177920	Serpina7	serine (or cysteine) peptidase inhibitor, clade A (alpha-1 antiproteinase, antitrypsin), member 7	-58.317136	1.15247E-07
XR_8722545	Gm39584	predicted gene, 39584, transcript variant X2	-54.292418	1.1762E-06
NM_001359281	Ptprn	protein tyrosine phosphatase, receptor type, N	-53.714568	1.85183E-07
NM_133888	Smpdl3b	sphingomyelin phosphodiesterase, acid-like 3B	-51.985118	5.29914E-08
NM_001039537	Lif	leukemia inhibitory factor	-50.370879	4.10836E-08
NM_001310604	Lgi2	leucine-rich repeat LGI family, member 2	-49.150791	1.67958E-07
NM_009252,	Serpina3n	serine (or cysteine) peptidase inhibitor, clade A, member 3N	-48.005025	1.0461E-07
NM_008012	Akr1b8	aldo-keto reductase family 1, member B8	-46.891055	5.44033E-08
NM_001085390	Dusp5	dual specificity phosphatase 5	-45.624538	1.41577E-07
NM_001301404	Serpina10	serine (or cysteine) peptidase inhibitor, clade A (alpha-1 antiproteinase, antitrypsin), member 10	-42.341393	1.20481E-07
NM_178679	Zfp365	zinc finger protein 365	-40.107732	3.44534E-07
NM_008381	Inhbb	inhibin beta-B	-38.226626	3.08345E-07
XR_003946862	Gm33721	predicted gene, 33721, transcript variant X2	-36.329715	5.03119E-06
NM_011313	S100a6	S100 calcium binding protein A6 (calcyclin)	-35.633771	2.1235E-07
NM_001347458	Egr2	early growth response 2	-34.066802	1.02689E-06
NM_001301295	Cidec	cell death-inducing DFFA-like effector c	-30.701730	4.84136E-06
NM_009778	C3	complement component 3	-30.684457	4.76295E-07
NM_001359214	Svop	SV2 related protein	-30.446032	4.97301E-06
NM_001146031	Nrcam	neuronal cell adhesion molecule	-30.168145	8.3444E-07
NM_009140	Cxcl2	chemokine (C-X-C motif) ligand 2	-28.690603	5.06112E-06
NM_027998	Cldn23	claudin 23	-28.561885	5.45535E-05

Supplementary Table 4. Genes downregulated in the kidneys of *Inmt* transgenic mice+high dose Voc-induced acute kidney injury (TG AKI) compared with wild-type mice+high dose Voc-induced AKI (WT AKI)

Transcript_ID	Gene_Symbol	Description	TGAKI/WTAKI.fc	TGAKI/WTAKI.raw.pval
NM_010235	Fosl1	fos-like antigen 1	-1228.463143	1.21583E-13
NM_023256	Krt20	keratin 20	-1091.792101	6.06318E-16
NM_001166631	Havcr1	hepatitis A virus cellular receptor 1	-420.230963	6.11162E-14
NM_001270511	Gm10352	predicted gene 10352	-346.434194	2.98403E-10
NM_001270510	Gm10256	predicted gene 10256	-342.210592	3.20208E-10
NM_001166384	Rbmy	RNA binding motif protein, Y chromosome	-326.020117	4.28078E-10
NM_001357493	Saa1	serum amyloid A 1	-245.950815	1.36552E-10
NM_001270514	Gm21693	predicted gene, 21693	-201.102407	6.59945E-10
NM_001270512	Gm3376	predicted gene 3376	-194.384511	9.86308E-09
NM_001270515	Gm21704	predicted gene, 21704	-169.260165	1.88188E-09
NR_003548	Sprr2g	small proline-rich protein 2G	-155.522213	2.25984E-09
NM_001039701	Il1rn	interleukin 1 receptor antagonist	-145.238825	1.37826E-09
NM_001270513	Gm21677	predicted gene, 21677	-143.342061	5.15504E-09
NM_001270516	Gm21708	predicted gene, 21708	-137.417923	6.63195E-09
NM_008491	Lcn2	lipocalin 2	-133.172602	8.2833E-11
NM_009264	Sprr1a	small proline-rich protein 1A	-104.259489	4.70307E-10
NM_008176	Cxcl1	chemokine (C-X-C motif) ligand 1	-80.491072	4.11054E-09
NM_001270518	Gm4064	predicted gene 4064	-78.939382	1.91082E-06
NM_008871	Serpine1	serine (or cysteine) peptidase inhibitor, clade E, member 1	-78.257134	2.4985E-09
NM_001291066	Adam8	a disintegrin and metallopeptidase domain 8	-72.569148	1.34814E-08
NM_001081185	Flnc	filamin C, gamma	-70.179308	6.3051E-09
NM_019450	Il1f6	interleukin 1 family, member 6	-62.079326	5.32259E-07
NM_177920	Serpina7	serine (or cysteine) peptidase inhibitor, clade A (alpha-1 antiproteinase, antitrypsin), member 7	-58.317136	1.15247E-07
XR_8722545	Gm39584	predicted gene, 39584, transcript variant X2	-54.292418	1.1762E-06
NM_001359281	Ptprn	protein tyrosine phosphatase, receptor type, N	-53.714568	1.85183E-07
NM_133888	Smpdl3b	sphingomyelin phosphodiesterase, acid-like 3B	-51.985118	5.29914E-08
NM_001039537	Lif	leukemia inhibitory factor	-50.370879	4.10836E-08
NM_001310604	Lgi2	leucine-rich repeat LGI family, member 2	-49.150791	1.67958E-07
NM_009252,	Serpina3n	serine (or cysteine) peptidase inhibitor, clade A, member 3N	-48.005025	1.0461E-07
NM_008012	Akr1b8	aldo-keto reductase family 1, member B8	-46.891055	5.44033E-08
NM_001085390	Dusp5	dual specificity phosphatase 5	-45.624538	1.41577E-07
NM_001301404	Serpina10	serine (or cysteine) peptidase inhibitor, clade A (alpha-1 antiproteinase, antitrypsin), member 10	-42.341393	1.20481E-07
NM_178679	Zfp365	zinc finger protein 365	-40.107732	3.44534E-07
NM_008381	Inhbb	inhibin beta-B	-38.226626	3.08345E-07
XR_003946862	Gm33721	predicted gene, 33721, transcript variant X2	-36.329715	5.03119E-06
NM_011313	S100a6	S100 calcium binding protein A6 (calcyclin)	-35.633771	2.1235E-07
NM_001347458	Egr2	early growth response 2	-34.066802	1.02689E-06
NM_001301295	Cidec	cell death-inducing DFFA-like effector c	-30.701730	4.84136E-06
NM_009778	C3	complement component 3	-30.684457	4.76295E-07
NM_001359214	Svop	SV2 related protein	-30.446032	4.97301E-06
NM_001146031	Nrcam	neuronal cell adhesion molecule	-30.168145	8.3444E-07
NM_009140	Cxcl2	chemokine (C-X-C motif) ligand 2	-28.690603	5.06112E-06
NM_027998	Cldn23	claudin 23	-28.561885	5.45535E-05
XR_383367	Gm32762	predicted gene, 32762, transcript variant X3	-28.400446	7.33181E-06
NM_001044384	Timp1	tissue inhibitor of metalloproteinase 1	-28.338830	1.57148E-06

Supplementary Table 5. Genes upregulated in kidneys of Regnase-2 conditional knockout mice

Transcript_ID	Gene_Symbol	Description	CKO/Control.fc	CKO/Control.raw.pval
gene-Gm38421	Gm38421	predicted gene, 38421	132.678944	1.8896E-63
NM_013701	Ugt1a2	UDP glucuronosyltransferase 1 family, polypeptide A2	92.991351	5.811E-115
NM_009252	Serpina3n	serine (or cysteine) peptidase inhibitor, clade A, member 3N	91.564693	2.2698E-57
NM_008491	Lcn2	lipocalin 2	91.313401	3.441E-101
NM_011333	Ccl2	chemokine (C-C motif) ligand 2	80.423902	1.1302E-77
NM_001081123	Arhgap36	Rho GTPase activating protein 36	51.836562	4.5837E-23
NM_001301404	Serpina10	serine (or cysteine) peptidase inhibitor, clade A (alpha-1 antiproteinase, antitrypsin), member 10	51.173182	7.7155E-81
NM_009778,	C3	complement component 3	50.091267	1.027E-110
NM_011426	Siglec1	sialic acid binding Ig-like lectin 1, sialoadhesin	48.513961	2.0863E-53
NM_008176	Cxcl1	chemokine (C-X-C motif) ligand 1	37.560261	1.027E-64
NM_177686,	Clec12a	C-type lectin domain family 12, member a	37.455560	4.1142E-35
NM_010935	Npy6r	neuropeptide Y receptor Y6	34.714917	1.6284E-38
NM_013468	Ankrd1	ankyrin repeat domain 1 (cardiac muscle)	32.443056	9.987E-65
NM_018827	Crlf1	cytokine receptor-like factor 1	31.499097	5.4247E-24
NM_001370743	Ltbp2	latent transforming growth factor beta binding protein 2	31.320136	1.7098E-40
NM_001025779	Cdc6	cell division cycle 6	27.711450	1.0194E-13
NM_021887,	Il21r	interleukin 21 receptor	27.068114	2.3663E-13
NM_001313963	Krt19	keratin 19	26.713420	2.7582E-60
NM_080457	Muc4	mucin 4	25.789041	2.3375E-31
NM_007895	Ear2	eosinophil-associated, ribonuclease A family, member 2	25.401940	1.5642E-20
NM_001313760	Tlr8	toll-like receptor 8	25.382025	2.5734E-26
NM_013590	Lyz1	lysozyme 1	25.072364	3.0378E-20
NM_008127	Gjb4	gap junction protein, beta 4	24.173100	4.728E-12
NM_001025610	Ms4a7	membrane-spanning 4-domains, subfamily A, member 7	23.277464	1.5935E-24
NM_011267	Rgs16	regulator of G-protein signaling 16	22.283338	1.6779E-25
NM_011338	Ccl9	chemokine (C-C motif) ligand 9	22.087655	5.2097E-31
NM_00116335	Inka2	inka box actin regulator 2	21.997836	3.6905E-25
gene-LOC115488377	LOC115488377	.	21.921423	6.5297E-11
NM_028216	Psca	prostate stem cell antigen	21.618775	3.3498E-29
NM_001357007	P2ry12	purinergic receptor P2Y, G-protein coupled 12	20.956419	2.4755E-10
NM_023137	Ubd	ubiquitin D	20.865272	2.0407E-44
NM_001347458	Egr2	early growth response 2	20.485716	1.8262E-33
NM_001190320	Clec4n	C-type lectin domain family 4, member n	20.458299	8.4606E-17
NM_029499,	Ms4a4c	membrane-spanning 4-domains, subfamily A, member 4C	20.284826	2.0755E-23
NM_133983	Cd276	CD276 antigen	20.284826	2.0755E-23
NM_001024230	Gm5431	predicted gene 5431	19.963935	2.483E-16
NM_001309637	Clec7a	C-type lectin domain family 7, member a	19.924667	7.8045E-41
NM_001302966	Gm21188	predicted gene, 21188	19.864278	1.1959E-13
NR_003520	Mx1	MX dynamin-like GTPase 1	19.799147	3.563E-16
NM_008240	Foxj1	forkhead box J1	19.224607	3.8675E-33
NM_026818	Cilp2	cartilage intermediate layer protein 2	18.338757	1.393E-12
NM_001204201	Spp1	secreted phosphoprotein 1	18.135558	3.4843E-72
NM_009264	Sprr1a	small proline-rich protein 1A	18.101981	2.4213E-29
NM_001304551	Stil	Scl/Tal1 interrupting locus	18.061405	5.8298E-09
NM_00108118	Flnc	filamin C, gamma	17.906620	6.4504E-30
NM_011090	Lilra6	leukocyte immunoglobulin-like receptor, subfamily A (with TM domain), member 6	17.739737	9.2043E-09

Supplementary Table 6. Genes downregulated in kidneys of Regnase-2 conditional knockout mice

Transcript_ID	Gene_Symbol	Description	CKO/Control.fc	CKO/Control.raw.pval
NR_033591	Gm6300	predicted gene 6300	-636.301700	5.339E-129
NM_001163032,	Synpr	synaptoporin	-39.688248	6.2308E-30
NM_001077425	Cntnap5a	contactin associated protein-like 5A	-36.757612	5.4161E-42
NM_144856	Slc22a7	solute carrier family 22 (organic anion transporter), member 7	-21.202409	2.4249E-69
XR_879464	Gm42060	predicted gene, 42060	-20.514812	2.5726E-44
NM_016870	Acsm3	acyl-CoA synthetase medium-chain family member 3	-20.087708	7.1606E-76
XR_373448	Gm4208	predicted gene 4208, transcript variant X2	-19.393998	7.4597E-57
XR_388069	Gm31037	predicted gene, 31037	-18.421680	5.9007E-15
XR_001778761	Gm31106	predicted gene, 31106	-17.400690	7.3544E-24
NR_151541	Gm12354	predicted gene 12354	-17.282960	3.0227E-07
NR_045760	0610031O16Rik	RIKEN cDNA 0610031O16 gene	-17.163430	1.8794E-32
NM_001359131	Mpped1	metallophosphoesterase domain containing 1	-16.782977	2.085E-46
NM_007825	Cyp7b1	cytochrome P450, family 7, subfamily b, polypeptide 1	-15.732784	5.641E-65
gene-Gm12978	Gm12978	predicted gene 12978	-15.582196	0.000187
NM_001109764,	Ctnna2	catenin (cadherin associated protein), alpha 2	-15.023169	3.3278E-27
NM_013797	Slco1a1	solute carrier organic anion transporter family, member 1a1	-14.485589	3.0821E-63
XR_003952881	LOC115489017	uncharacterized LOC115489017	-14.110889	1.6377E-11
NM_010594	Kap	kidney androgen regulated protein	-14.029302	2.1958E-62
NR_015580	Sox2ot	SOX2 overlapping transcript (non-protein coding)	-13.840251	8.426E-06
NR_045334	B930092H01Rik	RIKEN cDNA B930092H01 gene	-13.823503	2.5854E-11
NM_001128145	5830411N06Rik	RIKEN cDNA 5830411N06 gene	-13.574584	3.5989E-18
XM_030248232	Gm11100	predicted gene 11100	-13.188357	1.5251E-21
NM_001177900	Rd3	retinal degeneration 3	-11.805382	2.1117E-08
NM_001029937	Sec14l3	SEC14-like lipid binding 3	-11.766461	6.8483E-41
NM_007376	Pzp	PZP, alpha-2-macroglobulin like	-11.713567	5.8395E-54
NM_007606	Car3	carbonic anhydrase 3	-11.351613	1.3754E-44
NR_033546	Gm15348	predicted gene 15348	-11.334689	1.3374E-34
XR_001783259	9330198N18Rik	RIKEN cDNA 9330198N18 gene, transcript variant X2	-11.086084	0.00014282
NM_001081688	Tmprss9	transmembrane protease, serine 9	-11.005640	8.4014E-28
XR_877476	Gm41750	predicted gene, 41750	-10.732070	3.1919E-12
gene-LOC115487780	LOC115487780	.	-10.397542	0.00025279
XM_030255696	Gm40369	predicted gene, 40369, transcript variant X2	-10.131579	3.6972E-09
XR_003953859	LOC115489460	uncharacterized LOC115489460	-9.956349	1.5853E-10
gene-LOC115487418	LOC115487418	.	-9.709000	0.00044798
XR_003952551	Gm36003	predicted gene, 36003, transcript variant X3	-9.650278	9.2043E-09
XR_001784517	Gm13110	predicted gene 13110, transcript variant X1	-9.445384	4.9987E-05
NM_146013	Sec14l4	SEC14-like lipid binding 4	-9.304310	4.2092E-13
XR_003947599	Gm31774	predicted gene, 31774, transcript variant X3	-9.127933	1.7731E-35
XR_003948275	Gm34159	predicted gene, 34159, transcript variant X2	-9.020458	0.00079462
XR_380546	Gm33263	predicted gene, 33263, transcript variant X1	-8.952188	1.0567E-05
NM_001039554	Angptl7	angiopoietin-like 7	-8.887422	1.2353E-45
NM_008955	Rhox6	reproductive homeobox 6	-8.687676	9.198E-08
NR_045504	4933402C06Rik	RIKEN cDNA 4933402C06 gene	-8.471401	9.9993E-11
NM_001001320	Tbx10	T-box 10	-8.470810	1.4794E-23
NM_001081134	Kcng1	potassium voltage-gated channel, subfamily G, member 1	-8.382535	4.1844E-23
XR_003948829	Gm28905	predicted gene 28905, transcript variant X2	-8.331917	0.00141036
